# The single and combined effects of deltamethrin and polyethylene microplastics on the development and biochemical responses of *Xenopus laevis* in early life stages

**DOI:** 10.1007/s10646-026-03028-5

**Published:** 2026-01-23

**Authors:** Duygu Ozhan Turhan, Cihan Anıl Benli, Muhittin Yurekli, Abbas Güngördü

**Affiliations:** 1 Department of Biology, Faculty of Arts and Science, Inonu University, 44280 Malatya, Türkiye; 2 Laboratory of Environmental Toxicology, Department of Biology, Faculty of Arts and Science, Inonu University, Malatya, 44280 Türkiye

**Keywords:** Mixture toxicity, Developmental toxicity, FETAX assay, Oxidative stress, Enzymatic biomarkers

## Abstract

The co-occurrence of microplastics and pesticides in freshwater ecosystems raises concerns for aquatic organisms. However, their combined effects remain poorly understood. In this study, the single and combined effects of deltamethrin (DEL) and polyethylene microplastics (PE-MPs) were investigated in the early life stages of *Xenopus laevis*. Embryonic developmental toxicity was evaluated under standardized Frog Embryo Teratogenesis Assay–*Xenopus* (FETAX) conditions in embryos, and sublethal biochemical responses were also assessed in embryos and tadpoles. In the first phase, embryos were exposed for 96 h to a concentration range of DEL (3.125–6,400 µg/L) and PE-MPs (50–1,000 mg/L) to estimate the median lethal concentration (LC_50_) and the median effective concentration for malformation (EC_50_), as well as the teratogenic index (TI). In the second phase, assays were performed at DEL 2.72 and 13.6 µg/L (alone or combined with 100 mg/L PE-MPs) and PE-MPs 50 and 250 mg/L alone, assessing malformation and lethality in embryos and enzymatic biomarkers in both embryos and tadpoles. DEL caused significant embryotoxicity, with 96 h LC_50_ and EC_50_ values of 68.0 µg/L and 7.58 µg/L, respectively, and a TI of 8.97. PE-MPs alone did not induce lethality or malformations but caused biochemical alterations. Combined exposures produced additive (glutathione S-transferase, GST; carboxylesterase, CaE) and antagonistic (catalase, CAT; acetylcholinesterase, AChE) patterns; notably, CAT showed a synergistic pattern in embryos based on interaction index analysis. In tadpoles, 250 mg/L PE-MP concentration inhibited glutathione reductase (GR) and AChE activity, suggesting life stage–dependent sensitivity. These findings underscore the complexity of mixture toxicity and emphasize the need for combined exposure assessments in amphibian ecotoxicology.

**Supplementary Information:**

The online version contains supplementary material available at 10.1007/s10646-026-03028-5.

## Introduction

The term “plastic” refers to a broad class of synthetic polymers derived from petroleum sources, including polyethylene (PE), polystyrene, polypropylene, polyvinyl chloride, and nylon (Anderson et al. 2016). These materials are widely used due to their durability, low density, and low thermal conductivity (Meng et al. 2020). Since their industrial introduction in the 1940s, global plastic production has increased steadily, reaching approximately 400 million tons in 2022, with further growth projected by 2050 (PlasticsEurope 2023). PE is the most prevalent polymer, accounting for about 30% of global plastic demand, with annual production exceeding 140 million tons (Anderson et al. 2016; Houssini et al. 2025).

Plastic waste is pervasive in marine, freshwater, and terrestrial environments, where it degrades via mechanical, photochemical, and biological processes. These processes result in the formation of microplastics (MPs; particles < 5 mm) and nanoplastics (NPs; particles < 100 nm) (Cole et al. 2011). MPs can originate as primary particles, which are released directly from products such as cosmetics, textiles, and tire wear, or as secondary particles, which are formed by the fragmentation of larger plastic debris (Fang et al. 2019). PE-MPs are among the most frequently detected MPs in aquatic environments and display variability in size, shape, and color (Zhang et al. 2018). Due to their persistence and bioavailability, MPs can enter aquatic food webs and may adversely affect ecosystem integrity and potentially increase human exposure (Hasan et al. 2024; Tatlı et al. 2025; Timaná Morales et al. 2025).

Synthetic pyrethroids are a widely used class of insecticides applied in agriculture, veterinary medicine, public health, and domestic settings (El-Demerdash 2007). These compounds are chemically classified as Type I (non-cyano) or Type II (alpha-cyano) pyrethroids (Soderlund 2020). Their neurotoxic mode of action involves modulating sodium, potassium, GABA, and calcium channels, which disrupts nerve transmission and causes paralysis in target pests and non-target aquatic organisms (Ren et al. 2016; Yang et al. 2020). Pyrethroids are highly toxic to fish, amphibians, and other aquatic invertebrates, even at low environmental concentrations, while showing comparatively lower toxicity to birds and mammals (Brander et al. 2009; Hong et al. 2021; Ranatunga et al. 2023).

Deltamethrin (DEL), a type II pyrethroid, is used globally for crop protection, livestock farming, and vector control, including programs targeting disease vectors, such as mosquitoes (Lu et al. 2019; Yadav et al. 2001). Environmental monitoring reports have detected DEL concentrations ranging from a few micrograms per liter in surface waters to milligrams per liter near industrial discharges (Rodrigues et al. 2023). Due to its hydrophobic nature and low water solubility, deltamethrin tends to adsorb onto sediments, organic matter, and microplastic particles, which can affect its environmental fate and toxicity (Horton et al. 2018). Microplastics may act as vectors for hydrophobic organic pollutants, including pesticides, potentially modifying their transport, bioavailability, and toxicity (Trevisan et al. 2019; Wen et al. 2018; Xu et al. 2021). PE-MPs and DEL have been reported to co-occur in freshwater ecosystems (Luo et al. 2021; Soares et al. 2024), yet their combined toxicological effects on aquatic vertebrates remain insufficiently characterized.

Amphibians are particularly vulnerable to chemical pollution due to their permeable skin and life cycles that span both aquatic and terrestrial habitats (Fernandez et al. 2020). *Xenopus laevis* is a well-established ecotoxicological model widely used in developmental toxicity assays, including the Frog Embryo Teratogenesis Assay*–Xenopus* (FETAX; ASTM 2003), the Amphibian Metamorphosis Assay (AMA; OECD 2009), and the *Xenopus* Eleutheroembryo Thyroid Assay (OECD 2019). Both pesticides and microplastics are increasingly recognized as emerging contaminants in freshwater ecosystems that can co-occur and exert combined adverse effects on aquatic biota.

Recent studies have reported that microplastics can modify the bioavailability and toxicity of various environmental pollutants through adsorption or co-transport mechanisms (Sunny et al. 2025). For instance, co-exposure of MPs with metals, pharmaceuticals, or pesticides has been shown to induce oxidative stress, genotoxicity, and developmental alterations in amphibians and fish (Rahman et al. 2024; Subaramaniyam et al. 2023; Tang 2025; Zhang et al. 2023). Although mixture toxicity studies involving MPs and pesticides in amphibians remain limited, emerging evidence suggests that MPs can alter contaminant uptake and detoxification capacity – leading to species- and stage-specific responses (Tang 2025; Zhang et al. 2023).

In this context, the present study aimed to evaluate the lethal and sublethal effects of DEL and PE-MPs, both individually and in combination, on the early life stages of *X. laevis*. A three-tiered experimental design was employed. First, the FETAX assay was used to evaluate mortality, malformations, and developmental delays. Second, the interactive effects of sublethal DEL concentrations (LC_50_/25 and LC_50_/5) alone and in combination with 100 mg/L PE-MPs were evaluated. Third, key enzymatic biomarkers involved in detoxification, oxidative stress, and neurotoxicity, including glutathione S-transferase (GST), glutathione reductase (GR), catalase (CAT), carboxylesterase (CaE), and acetylcholinesterase (AChE), were measured in embryos and tadpoles to elucidate physiological responses. This integrated approach provides novel insights into the complex interactions between pesticide and microplastic mixtures, enhancing our understanding of the ecological risks these contaminants pose to amphibian populations in freshwater environments.

## Materials and methods

### Chemicals and reagents

Virgin spherical polyethylene (PE) particles (40 μm diameter, 434272, Sigma-Aldrich, USA) and deltamethrin (DEL; Boston BioProducts, BDA-275575, USA) were tested in this study. Morphological verification of the PE-MPs was performed using light microscopy, as the particles were virgin, commercially standardized, and pre-characterized by the supplier. Particle diameters were measured using calibrated ImageFocus software, and descriptive parameters (mean ± SD, median, min–max, D10, D90) were summarized (Supplementary Table S1). Representative frequency histograms and images illustrating morphology and dispersion/aggregation behavior in the medium were provided (Supplementary Figures S3–S4). Additional reagents for biochemical assays, including 1-chloro-2,4-dinitrobenzene (CDNB), 5,5′-dithio-bis(2-nitrobenzoic acid) (DTNB), p-nitrophenyl acetate (PNPA), acetylthiocholine iodide (ACTI), reduced glutathione (GSH), and bovine serum albumin (BSA), were obtained from Sigma-Aldrich. Oxidized glutathione (GSSG) and NADPH were sourced from MP Biomedicals (USA).

### Test organisms

Adult *Xenopus laevis* were obtained from the Aquatic Vertebrates Experimental Unit at Inonu University and maintained at 23 ± 1 °C with a 12 h photoperiod. The FETAX solution used as the control was prepared according to ASTM Type I water standards (ASTM 2003), with the specified salts dissolved in one liter of distilled water. Adult frogs were acclimated for two days prior to hormonally induced breeding using human chorionic gonadotropin (hCG; Chorulon^®^, MSD, Germany; 600 IU for males and 500 IU for females). The fertilized eggs were then transferred to an aerated FETAX solution for exposure. Embryos at Nieuwkoop and Faber stages 8–11 were used for toxicity testing and biochemical assays. Tadpoles were maintained until stage 46 for subsequent biochemical analyses. All animal procedures were approved by the Inonu University Local Ethics Committee for Animal Experiments (Protocol No. 13505) and conducted in accordance with ASTM E1439-98 (ASTM 2003).

### FETAX assay for single substances

The FETAX assay was conducted to determine the LC_50_, EC_50_, the minimum concentration to inhibit growth (MCIG), and the teratogenic index (TI) for DEL and PE-MP. DEL concentrations (3.125–6,400 µg/L) were based on preliminary range-finding and literature to cover both environmentally relevant and high-end exposure conditions, as reported in surface waters and near agricultural runoff (Rodrigues et al. 2023; Shi et al. 2024). This range ensured adequate coverage for accurate LC_50_ and TI derivation.

The PE-MP concentrations (50–1,000 mg/L) were selected based on studies of freshwater fish that reported oxidative stress at similar levels (de Souza Freire et al. 2023; Lee et al. 2023), despite the lack of *X. laevis*–specific data and were used as high-end laboratory concentrations to support mechanistic assessment of sublethal responses and potential thresholds.

DEL stock solution was dissolved in dimethyl sulfoxide (DMSO) and diluted in FETAX; the final DMSO concentration did not exceed 0.006% (v/v). PE-MP solutions were prepared directly in FETAX. The pH, conductivity, and temperature of the FETAX solution used in the study were 7.78 ± 0.04, 1.54 ± 0.01 mS/cm, and 22 ± 0.2 °C, respectively. Embryos (stages 8–11) were exposed in 24-well plates (four embryos per well). Each treatment used 32 embryos (eight wells), while controls used 48 embryos (12 wells). Solutions were replaced every 48 h, and the embryos were examined every 24 h throughout the 96 h exposure period. The surviving embryos were euthanized with MS222 (200 mg/L), fixed in 3% formalin, and photographed for morphological assessment. Lengths were measured using Euromex ImageFocus 4.0 software.

### FETAX assay for mixtures

Mixture tests used two DEL concentrations (LC_50_/25: 2.72 µg/L and LC_50_/5: 13.6 µg/L) selected as sublethal, monitoring-relevant concentrations for mechanistic assessment, allowing for sufficient survival and sublethal endpoint assessment (Rodrigues et al. 2023; Shi et al. 2024). The PE-MP concentrations (50, 100, and 250 mg/L) included levels that were previously shown to cause oxidative stress. Mixture groups included each DEL concentration with 100 mg/L PE-MP. The methods mirrored those used for single-substance testing.

### Biochemical analyses

Biochemical assessments were performed on *X. laevis* embryos (stages 8–11) and tadpoles (stage 46) exposed to PE-MPs (50, 100, or 250 mg/L), DEL (2.72–13.6 µg/L), or mixtures of the two (100 mg/L PE-MPs + 2.72 µg/L or 13.6 µg/L DEL). The concentrations were selected based on developmental toxicity results and environmentally relevant sublethal levels. Fifteen individuals per replicate (five replicates per group) were exposed to a 10 mL test solution in 25 mL plastic containers. The solutions were renewed every 48 h, and the embryos were examined at 24 h intervals throughout the 96 h exposure period. Survivors were euthanized with buffered MS222 (200 mg/L), transferred to microcentrifuge tubes on ice, and stored at -80 °C until biochemical analysis.

The samples were homogenized on ice in a 0.1 M potassium phosphate buffer solution (pH 7.4) containing 150 mM KCl, 1 mM EDTA, and 0.05 mM DTT. The homogenates were then centrifuged at 16,000 g for 20 min at 4 °C. The resulting supernatants were used immediately for enzyme assays. Enzyme activities were measured for GST, GR, CAT, CaE, and AChE spectrophotometrically using established methods with slight modifications (Güngördü and Turhan 2024). GST activity was assessed by CDNB conjugation at 344 nm (Habig et al. 1974). GR activity was monitored via DTNB reduction at 405 nm (Stephensen et al. 2000). CAT activity was determined by measuring H₂O₂ decomposition at 240 nm (Aebi 1974). CaE activity was measured using a PNPA substrate adapted for microplate readers (Santhoshkumar and Shivanandappa 1999). AChE activity was quantified using the ACTI substrate by adapting the method of Ellman et al. (1961) for microplates (Ozmen et al. 1998). Total protein content was measured using the Bradford assay (Bradford 1976) to normalize the enzyme activities.

### High-performance liquid chromatography (HPLC) analysis

Before exposure, DEL concentrations in the test media were verified using HPLC (1100 System, Agilent Technologies, USA) for biochemical assays. Analyses were conducted with a C18 reversed-phase column (5 μm, 4.6 × 250 mm) at 25 °C using a mobile phase of acetonitrile and distilled water (80:20, v/v) (Mat Hussin et al. 2021) at a flow rate of 1.0 mL/min. The injection volume was 10 µL, and detection was performed at 230 nm. Quantification was based on a calibration curve (1.56–400 µg/L, R² = 0.994). Representative chromatograms and measured DEL concentrations are available in Supplementary Material (Table S2 and Figures S1).

### Statistical analysis and calculations

The 96 h LC_50_ and EC_50_ values for DEL were calculated using Finney’s probit analysis with the U.S. EPA Probit Analysis Program (version 1.5). The TI was determined as the ratio of LC_50_ to EC_50_.

All statistical analyses were performed using GraphPad Prism 8 (GraphPad Software, USA). Normality and homogeneity of variances were assessed using Kolmogorov–Smirnov and Bartlett’s tests, respectively. Parametric data were analyzed using a one-way ANOVA followed by a Dunnett’s post hoc test to compare treatments with controls. Nonparametric data were analyzed using the Kruskal–Wallis test, followed by the Dunn multiple comparison test. Statistical significance was set at *p* < 0.05.

In addition, pairwise comparisons among treatment groups (e.g., between single and mixture exposures) were performed using independent t-tests for normally distributed data or Mann–Whitney U tests for nonparametric data. These additional analyses allowed for a more detailed assessment of specific differences between DEL, PE-MP, and DEL + PE-MP groups at corresponding concentrations.

Statistical power and sample size justification were assessed a priori; detailed power analysis is provided in the Supplementary Materials.

To evaluate the interaction between DEL and PE-MPs in mixtures, we calculated the interaction index (II) following Peluso et al. (2024):*​ II = (M + Co)/(A1 + A2).*

*M* is the mean biomarker response in the mixture group; *A1* and *A2* are the means of the single-exposure groups (DEL and PE-MPs); and *Co* is the control mean. For biomarkers that are increased relative to the control group, an *II* value greater than 1 indicates synergism and an II value less than 1 indicates antagonism. For biomarkers that are decreased relative to the control group, an II value greater than 1 indicates antagonism and an II value less than 1 indicates synergism. Values of II between 0.95 and 1.05 were considered additive. Only mixtures that caused statistically significant differences in biomarkers or in which at least one treatment differed significantly from the control were considered for II analysis.

## Results

### Toxicity of individual DEL and PE-MP exposures

**Fig. 1:**
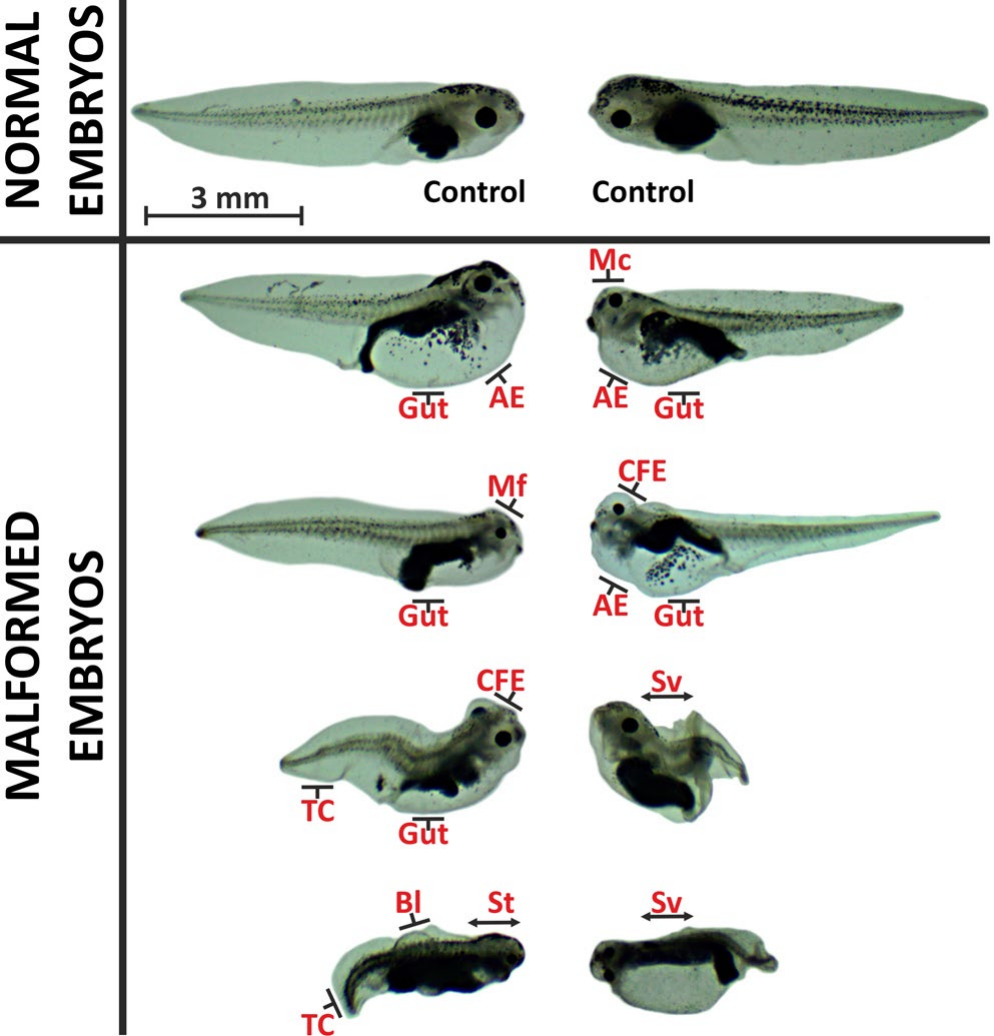
Types of malformations observed in *X. laevis* embryos exposed to the test substances. Malformations: Sv, severe (multiple, severe malformations); St, stunted growth; Gut, gut abnormalities (improper gut coiling and long, loosely coiled gut); AE, abdominal edema; CFE, craniofacial edema (face, head, and eye edema); Mc, microcephaly; Mf, microphthalmia; TC, tail curvature; Bl, blister

**Fig. 2:**
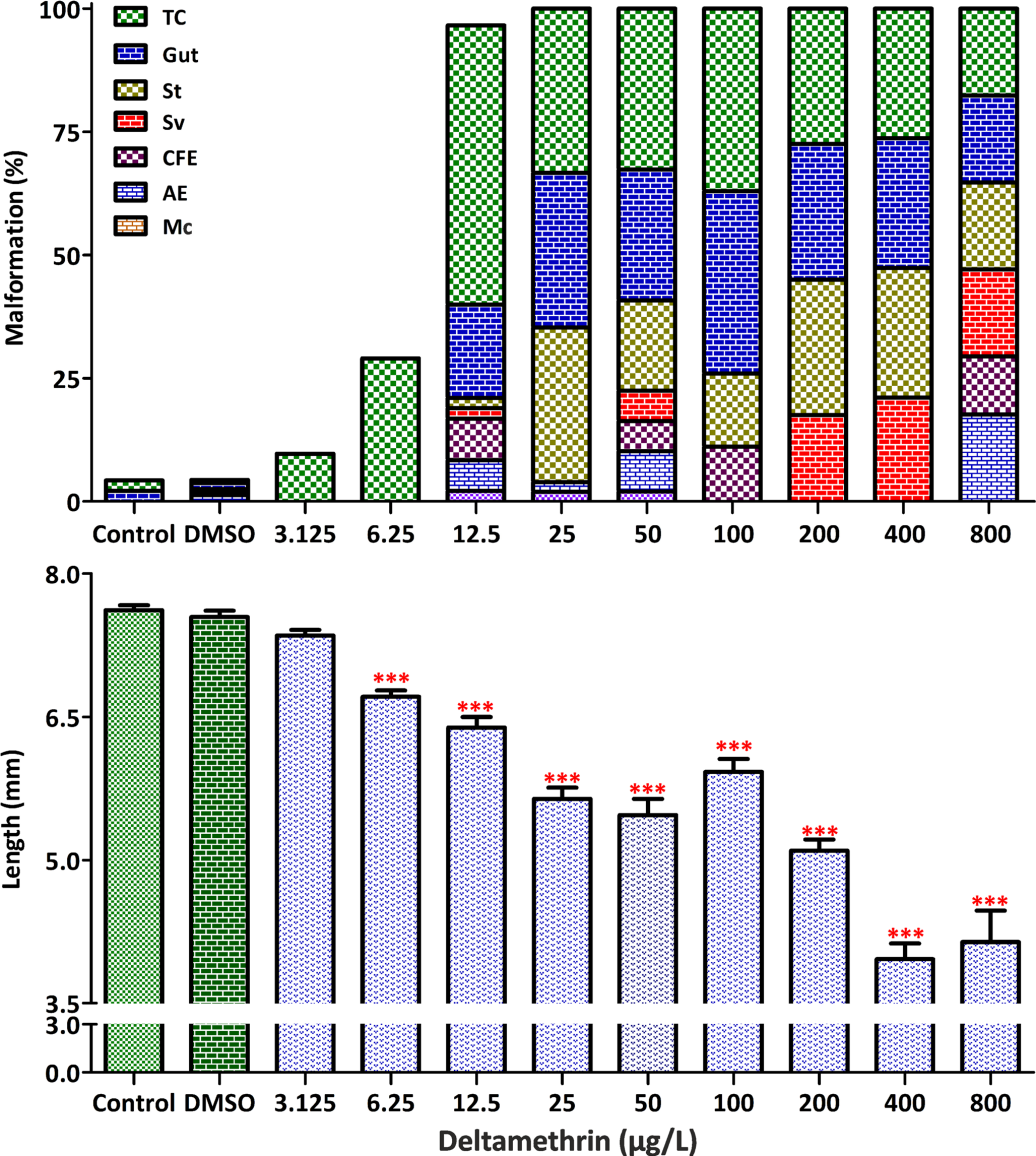
Malformations (%) and body length in embryos exposed to deltamethrin (DEL) concentrations for 96 h under FETAX (see also Table S3). Malformations as defined in Fig. 1. Bars show mean ± SE (*n* = 8 wells × 4 embryos/well). *p < 0.001 compared with control (one-way ANOVA with Dunnett’s or Kruskal–Wallis with Dunn’s, as appropriate)

According to the FETAX assay results, the 72 h and 96 h LC_50_ values for DEL were 2,035 µg/L and 68 µg/L, respectively (Table 1). The 96 h TI was calculated as 8.97, with an EC_50_ of 7.58 µg/L and an MCIG of 3.125 µg/L. Among the surviving embryos exposed to DEL, 67% exhibited malformations. The types of malformations and abbreviations used are defined in Fig. 1. The most frequent malformation types included tail curvature (65%), gut abnormalities (43%), and growth retardation (31%) (Fig. 2; see also Table S3 and Figure S2 in the Supplementary Material for details). Growth inhibition became statistically significant at concentrations ≥ 6.25 µg/L (*p* < 0.001).

**Table 1:** LC_50_, EC_50_, TI and MCIG values of deltamethrin for *X. laevis* embryos

Conc. (µg/L)	Conc. #	LC_50_ (95% CI) (µg/L)		EC_50_ (95% CI) (µg/L)	TI (LC_50_/EC_50_)	MCIG (µg/L)
		72 h	96 h			
3.125–6400	12	2035 (1397–3230)	68.00 (48.95–94.90)	7.58 (7.02–8.17)	8.97	3.125

LC_50_ = median lethal concentration; EC_50_ = median effective concentration for malformation; TI = teratogenic index, calculated as LC_50_/EC_50_; MCIG = minimum concentration to inhibit growth. Data are expressed with 95% confidence intervals

In contrast, exposure to PE-MPs alone resulted in ≤ 3% mortality and a total malformation rate of ≤ 4% across all tested concentrations. PE-MPs were verified by light microscopy; the particles were virgin, commercially standardized, and pre-characterized by the supplier. The mean particle diameter was ~ 41 μm (range 17–77 μm), and the particles appeared spherical and relatively smooth under light microscopy (Supplementary Table S1, Figures S3–S4). Consequently, LC_50_, EC_50_, and TI values could not be determined. No concentration-dependent increase in malformation frequency or growth inhibition was observed in PE-MP-treated embryos (see also Table S4 and Figure S5 in the Supplementary Material for details).

### Toxicity of DEL and PE-MP mixtures

**Fig. 3:**
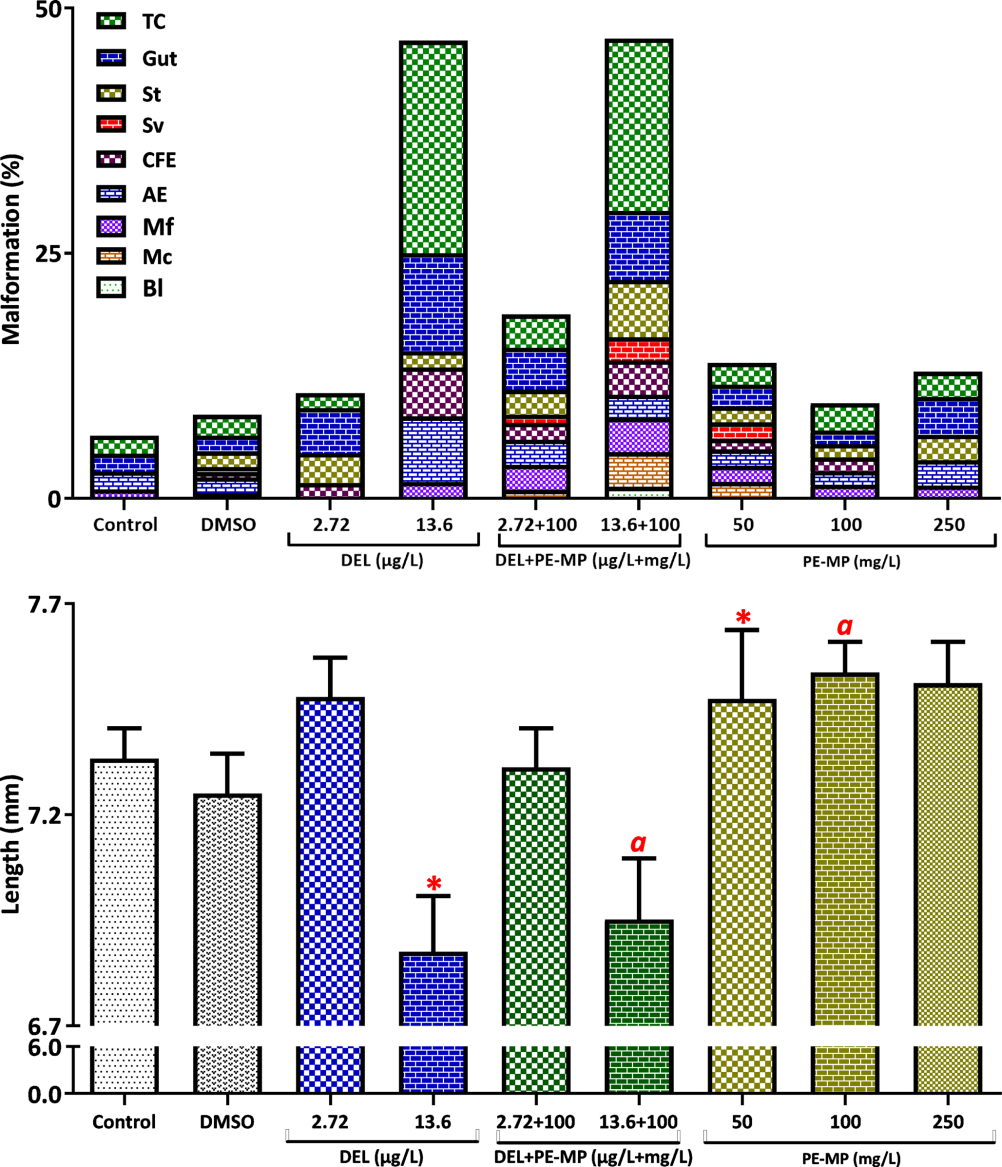
Malformations (%) and body length in embryos exposed to sublethal DEL and polyethylene microplastics (PE-MPs) alone and as DEL/PE-MP mixtures (96 h, FETAX; see also Table S5). Malformations as defined in Fig. 1. Data are mean ± SE. *p < 0.05 compared with control. Letters denote significant pairwise differences between treatment groups (*p* < 0.05; t-test or Mann–Whitney U, as applicable)

Mixture tests combined two sublethal DEL concentrations (2.72 and 13.6 µg/L) with three PE-MP concentrations (50, 100, and 250 mg/L). Fixed-combination groups were also tested using 100 mg/L PE-MP with each DEL concentration. Embryo viability remained high across all groups, ranging from 88% to 98% (see also Table S5 in the Supplementary Material for details.) Malformations were observed in 29% of embryos treated with DEL alone and in 33% of embryos exposed to mixtures. Significant growth inhibition occurred only in the 13.6 µg/L DEL group (*p* < 0.05), while the corresponding mixture group exhibited a similar but non-significant trend (Fig. 3; see also Table S5 and Figure S6 in the Supplementary Material for details).

**Fig. 4:**
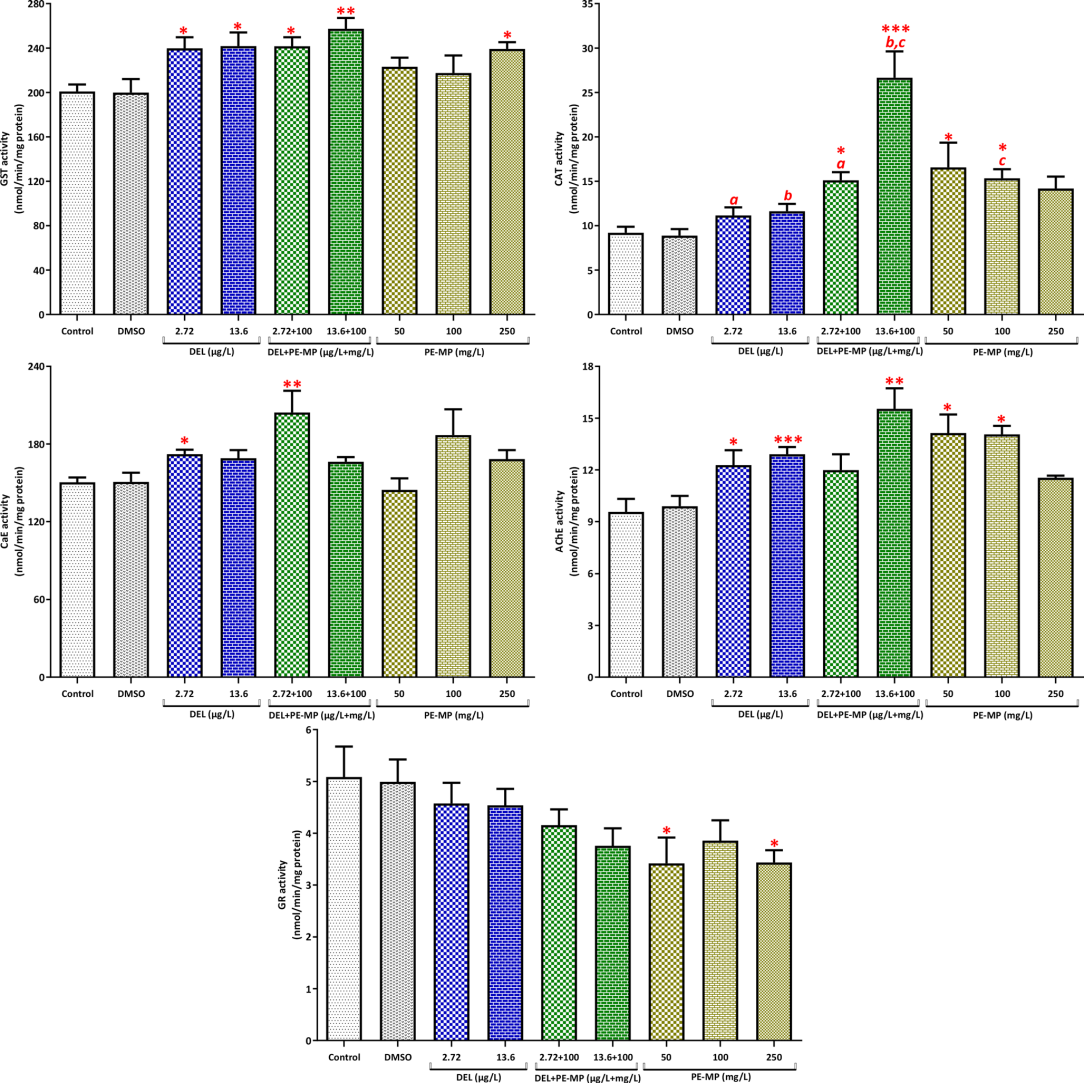
Biomarker responses (GST, GR, CAT, CaE, AChE) in embryos (stages 8–11; 96 h exposure) (see also Table S6). Values are mean ± SE of *n* = 5 biological replicates (each replicate = pool of 15 embryos). *, **, *** indicate *p* < 0.05, *p* < 0.01, *p* < 0.001 compared with control (one-way ANOVA/Dunnett’s or Kruskal–Wallis/Dunn’s). Letters denote significant pairwise differences between treatment groups (*p* < 0.05; t-test or Mann–Whitney U)

Embryos exposed to DEL alone or to the DEL/PE-MP mixtures exhibited a significant increase in GST activity compared to the control group (*p* < 0.05, Fig. 4; see also Table S6 in the Supplementary Material for details). However, pairwise comparisons showed no significant difference between the single and mixture groups for GST activity. Exposure to DEL alone did not significantly alter CAT activity (*p* > 0.05). However, exposure to the DEL/PE-MP mixtures or PE-MP alone (50 and 100 mg/L) significantly increased CAT activity. Pairwise tests showed that CAT in the 2.72 µg/L DEL + 100 mg/L PE-MP mixture was significantly higher than in 2.72 µg/L DEL alone, and CAT in the 13.6 µg/L DEL + 100 mg/L PE-MP mixture was higher than both 13.6 µg/L DEL and 100 mg/L PE-MP. Exposure to DEL alone increased AChE activity, and the increase was significant at 13.6 µg/L of DEL, both alone and in combination with PE-MP (*p* < 0.05). CaE activity increased significantly (*p* < 0.05) in embryos exposed to 2.72 µg/L DEL alone and in those exposed to the mixture of 2.72 µg/L DEL with PE-MP. Interaction index (II) analysis revealed that the low DEL mixture (2.72 µg/L) produced additive effects on GST and CaE and antagonistic effects on CAT and AChE in embryos. At the higher DEL level (13.6 µg/L), GST responses remained additive, CAT exhibited a synergistic pattern, and AChE showed antagonism (Table 2).

**Table 2:** Interaction indices (II) for biochemical biomarkers in embryos and tadpoles

	Embryos				Tadpoles			
	DEL	PE-MP	DEL	PE-MP	DEL	PE-MP	DEL	PE-MP
**Biomarkers**	2.72 µg/L	100 mg/L	13.6 µg/L	100 mg/L	2.72 µg/L	100 mg/L	13.6 µg/L	100 mg/L
**GST**	0.97	^***Ad***^	1.00	^***Ad***^	0.96	^***Ad***^	0.90	^***An***^
**GR**	–		–		–		–	
**CAT**	0.92	^***An***^	1.33	^***Si***^	0.74	^***An***^	0.77	^***An***^
**CaE**	0.99	^***Ad***^	–		0.95	^***Ad***^	–	
**AChE**	0.82	^***An***^	0.93	^***An***^	–		–	

^*Ad*^: Additive effect, ^*Si*^: Synergistic effect, ^*An*^: Antagonistic effect

**Fig. 5:**
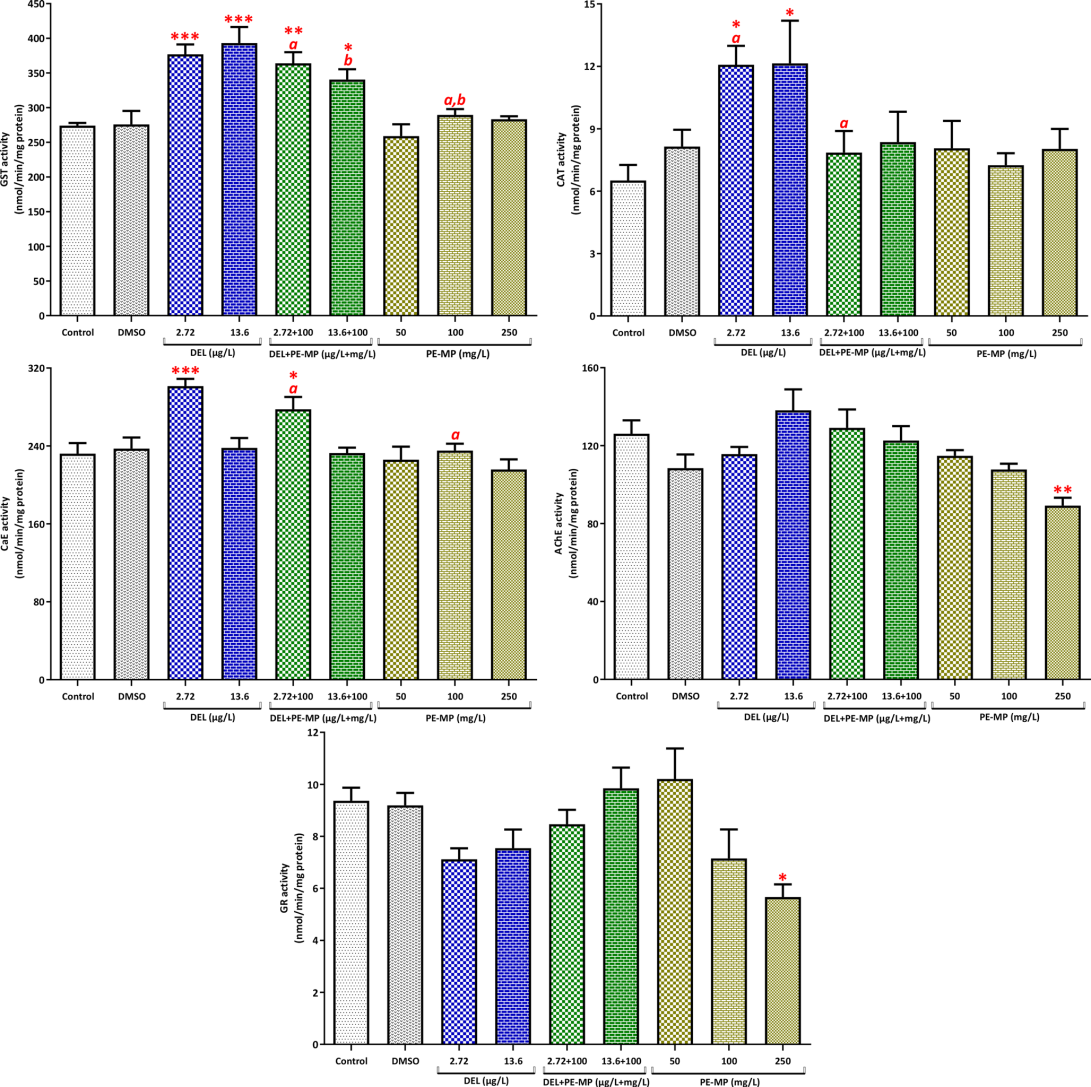
Biomarker responses (GST, GR, CAT, CaE, AChE) in tadpoles (stage 46; 96 h exposure) (see also Table S7). Values are mean ± SE of *n* = 5 biological replicates (each replicate = pool of 15 tadpoles). *, **, *** indicate *p* < 0.05, *p* < 0.01, *p* < 0.001 compared with control (one-way ANOVA/Dunnett’s or Kruskal–Wallis/Dunn’s). Letters denote significant pairwise differences between treatment groups (*p* < 0.05; t-test or Mann–Whitney U)

Similar biomarker trends were observed in tadpoles. DEL alone and DEL/PE-MP mixtures significantly increased GST activity (Fig. 5; see also Table S7 in the Supplementary Material for details). However, CAT activity was elevated only in the groups exposed to DEL alone, which differs from the embryonic pattern. CaE activity increased significantly in tadpoles exposed to 2.72 µg/L DEL (*p* < 0.05), and pairwise comparisons showed a significant difference between the DEL (2.72 µg/L) + PE-MP (100 mg/L) mixture and the single PE-MP (100 mg/L) group, suggesting that DEL modified the microplastic-induced response. PE-MP alone at 250 mg/L significantly inhibited AChE and GR activity. Tadpole analysis indicated additive interactions for GST and CaE and antagonistic interactions for CAT at the lower DEL concentration (Table 2). At the higher DEL concentration, both GST and CAT exhibited antagonistic responses.

## Discussion

The primary aim of this study was to evaluate the lethality and teratogenicity of DEL and PE-MPs in *X. laevis* embryos individually, and to assess their combined effects in the second phase. The tested PE-MP concentrations did not cause significant increases in mortality or malformations compared to the control group. This observation is consistent with their relatively inert morphology and size distribution, as characterized in this study. Light microscopy images also showed partial aggregation of PE-MPs in FETAX medium, which may influence their interaction with DEL and its apparent bioavailability (Supplementary Figure S4). This finding is consistent with previous research showing no lethality or phenotypic changes in *X. laevis* embryos exposed to microplastics at concentrations of up to 100 mg/L in FETAX assays (Bonfanti et al. 2021).

It is worth noting that the PE-MP concentrations used in this study (50–250 mg/L) exceed typical environmental levels, which are usually within the µg/L to low mg/L range. However, these levels were selected to simulate worst-case exposure conditions, as is common in laboratory ecotoxicology, and to reveal potential biochemical and developmental thresholds that could manifest under chronic or hotspot contamination scenarios. Accordingly, these concentrations are intended for mechanistic assessment and should not be interpreted as environmentally representative exposures.

Embryos exposed to DEL exhibited no lethality during the initial 48 h. However, LC_50_ values decreased significantly at 72 and 96 h (2,035 and 68 µg/L, respectively), indicating time-dependent toxicity associated with critical developmental stages, such as organogenesis and neurophysiological activation. This aligns with reports of pyrethroid-induced neurodevelopmental disruption (Andersen et al. 2022). Reported 96 h LC₅₀ values for DEL vary widely across species and life stages. *X. laevis* tadpoles exhibit values of 190 µg/L (pure) and 6.26 µg/L (commercial formulation) (Aydin-Sinan et al. 2012; Channing 1998), *Bufo arenarum* tadpoles exhibit values of approximately 4.4–4.5 µg/L (Salibián 1992), and juvenile fish as low as 1.5–1.9 µg/L (Srivastav et al. 1997; Vijayavel and Balasubramanian 2007). The intermediate LC_50_ observed in the present study in embryos underscores the influence of exposure conditions and formulations on toxicity and the sensitivity of different developmental stages.

The FETAX assay has been widely used to evaluate the toxic and teratogenic effects of environmental pollutants on amphibian embryos (Arancio et al. 2019; Cardoso-Vera et al. 2017; Menegola et al. 2024). This assay can quantify the teratogenicity index (TI), where values ≥ 1.5 indicate teratogenic potential according to ASTM standards. In this study, DEL was classified as teratogenic with a TI of 8.97, whereas PE-MP was not. Embryos exposed to DEL exhibited three main malformations: tail curvature, intestinal abnormalities, and stunted growth. Similar malformations have been reported in fish embryos exposed to DEL, indicating conserved developmental effects across vertebrate species (Kuder and Gundala 2018). Tail malformations in *X. laevis* embryos exposed to various pesticides have been linked to muscle overload and prolonged contractions (Yu et al. 2013).

Biochemically, exposure to DEL alone or in combination with PE-MP increased AChE activity in embryos. The lack of AChE inhibition is expected because AChE is not a direct target of DEL (Ray and Fry 2006). Pyrethroids act by binding to voltage-gated sodium channels, which causes prolonged nerve stimulation and neurotoxicity rather than directly inhibiting AChE (He et al. 2008). CaE activity significantly increased in embryos exposed to a low DEL/PE-MP mixture and in tadpoles exposed to DEL alone or in combination with PE-MP. CaEs play a role in detoxifying ester-containing pesticides, such as pyrethroids, and are sensitive biomarkers of environmental pesticide exposure (Wheelock et al. 2005; Yang et al. 2020). The increased CaE activity likely reflects phase I biotransformation and detoxification processes (Satoh and Hosokawa 2006).

GST, a key phase II detoxification enzyme and a widely used biomarker of xenobiotic exposure, was elevated in embryos and tadpoles following exposure to DEL alone or DEL/PE-MP mixtures (Anila et al. 2021; Lajmanovich et al. 2019; Pimpão et al. 2007; Tierbach et al. 2018). GST induction may represent an adaptive response that enables organisms to counteract oxidative and chemical stress (Liu et al. 2006).

CAT activity increased in embryos exposed to DEL/PE-MP mixtures or PE-MP alone; however, in tadpoles, CAT elevation occurred only with DEL exposure. DEL toxicity is often associated with oxidative stress due to the generation of reactive oxygen and nitrogen species, which disrupt metabolism and cellular defenses (Hafsi et al. 2024; Lu et al. 2019). The elevated GST and CAT activities, combined with the observed malformations, suggest that considerable oxidative stress may contribute to teratogenic effects. Differences in antioxidant enzyme responses between embryos and tadpoles may be explained by developmental changes in glutathione metabolism.

During early embryogenesis of *X. laevis*, antioxidant defense primarily depends on catalase (CAT) and superoxide dismutase (SOD) activities, while the glutathione-dependent system (including GST and GR) becomes more prominent after stage 35/36 as differentiation progresses (Rizzo et al. 2007). Accordingly, the moderate increase of phase I (CaE) and phase II (GST) enzyme activities observed in tadpoles compared with embryos may reflect the higher metabolic capacity and detoxification potential that develops during post-embryonic stages of *X. laevis*, rather than a fully adaptive compensatory response (Angelucci et al. 2002; Menon and Rozman 2007; Prokić et al. 2019). This stage-dependent antioxidant shift may involve transcriptional control of redox-responsive genes. In *X. laevis*, activation of the Nrf2 transcription factor under oxidative stress up-regulates glutathione S-transferase (GST) and aldo-keto reductase (AKR) isoforms, indicating that Nrf2-mediated regulation contributes to enhanced detoxification capacity during post-embryonic development (Malik and Storey 2009).

Only the highest PE-MP concentration (250 mg/L) significantly inhibited GR and AChE activities in tadpoles. AChE inhibition leads to acetylcholine accumulation, causing neurotoxicity and potentially lethality (Kaushal et al. 2021; Storck et al. 2025). GR maintains the critical glutathione redox balance for managing oxidative stress (van der Oost et al. 2003). Thus, inhibition of these enzymes reflects the sublethal toxic effects of PE-MP.

Analysis of the sublethal effects of DEL and PE-MP mixtures revealed mostly additive or antagonistic interactions across biomarkers. However, pairwise comparisons indicated that CAT activity in embryos exposed to DEL + PE-MP mixtures was significantly higher than in either single exposure, supporting an endpoint-specific synergistic pattern in CAT activity. Similarly, in tadpoles, a significant pairwise difference in CaE activity between the DEL (2.72 µg/L) + PE-MP (100 mg/L) mixture and the single PE-MP (100 mg/L) group suggested that DEL modified the microplastic-induced detoxification response. These results suggest that microplastics may modify the apparent toxicity of hydrophobic compounds like DEL, particularly through oxidative and detoxification-related pathways.

Similar mixture-dependent effects have been described in recent studies involving microplastics and other pollutants. For instance, Ye et al. (2025) demonstrated that co-exposure to polyethylene microplastics and bisphenol A induced synergistic endocrine disruption and cellular toxicity in zebrafish and MLTC-1 cell models, while Kim et al. (2024) reported developmental abnormalities and oxidative stress in zebrafish embryos exposed to mixtures of microplastics and plastic additives. Together with our data, these studies indicate that microplastics can modulate DEL bioavailability and redox responses, highlighting their capacity to alter oxidative and detoxification mechanisms in aquatic vertebrates.

Although the present experimental design ensured adequate power to detect medium-to-large effects, it may have been insufficient to capture subtle biochemical changes, particularly for variable biomarkers such as GR, CAT, and AChE. Embryonic assays showed slightly higher variability (CV up to ~ 26%) than tadpole assays, which may have increased the likelihood of Type II errors at low concentrations. Therefore, non-significant outcomes at the lowest doses should be interpreted with caution and supported by trends and effect-size considerations rather than p-values alone. In addition, higher-resolution imaging and more advanced microplastic characterization (e.g., polymer verification and surface/morphology analyses) would strengthen future studies.

These results highlight the importance of considering life stage and chemical combinations when assessing environmental risk, as mixture interactions can mask early cellular damage and lead to delayed toxicity (Burić et al. 2023). Combined exposure to MPs and pesticides has been shown to impair metamorphosis, reduce growth and fecundity, and alter behavioral responses in amphibians and fish (Rahman et al. 2024; Relyea 2009; Subaramaniyam et al. 2023). Such sublethal effects, even in the absence of acute mortality, may threaten amphibian population stability and disrupt freshwater ecosystem balance (Mann et al. 2009; Peluso et al. 2024).

## Conclusions

The PE-MP concentrations tested in this study (50–250 mg/L in mixtures; 50–1,000 mg/L in single exposures) did not induce significant lethality, malformations, or developmental delays in *X. laevis* embryos, indicating no observable acute developmental effects under the tested conditions. In contrast, even low concentrations of DEL caused significant embryotoxic and teratogenic effects, supporting its potency as a developmental toxicant. Sublethal DEL exposure also modulated the activities of several enzymes involved in the oxidative stress response and detoxification, such as GST and CAT. Distinct biochemical patterns were observed between embryonic and larval stages. Mixture effects were predominantly additive or antagonistic, with synergy limited to specific endpoints (e.g., CAT in embryos). Our results underscore the importance of considering pollutant mixtures in ecotoxicological risk assessments, as microplastics can modify the effects of co-occurring toxicants in ways that may be biologically relevant depending on life stage and endpoint. Given the growing likelihood of pesticide–microplastic co-occurrence in natural waters, such interactions warrant further investigation to better inform ecological risk assessments.

## Supplementary Information

Below is the link to the electronic supplementary material.

Supplementary Material 1

## Data Availability

No datasets were generated or analysed during the current study.

## References

[CR1] Aebi H (1974) Catalase. In: Bergmayer HU (ed) Methods of enzymatic analysis. Academic, London 673–684

[CR2] Andersen HR, David A, Freire C et al (2022) Pyrethroids and developmental neurotoxicity - A critical review of epidemiological studies and supporting mechanistic evidence. Environ Res 214:113935 10.1016/j.envres.2022.113935 35870501 10.1016/j.envres.2022.113935

[CR4] Anderson JC, Park BJ, Palace VP (2016) Microplastics in aquatic environments: implications for Canadian ecosystems. Environ Pollut 218:269–280 10.1016/j.envpol.2016.06.074 27431693 10.1016/j.envpol.2016.06.074

[CR6] Angelucci S, Sacchetta P, De Luca A et al (2002) Glutathione transferase isoenzymes from frog (*Xenopus laevis*) liver and embryo. Biochim Biophys Acta Gen Subj 1569(1):81–85 10.1016/S0304-4165(01)00238-0 10.1016/s0304-4165(01)00238-0 11853961

[CR8] Anila PA, Sutha J, Nataraj D et al (2021) *In vivo* evaluation of nano-palladium toxicity on larval stages and adult of zebrafish (*Danio rerio*). Sci Total Environ 765:144268 10.1016/j.scitotenv.2020.144268 33418331 10.1016/j.scitotenv.2020.144268

[CR10] Arancio AL, Cole KD, Dominguez AR et al (2019) Bisphenol A, bisphenol AF, di-n-butyl phthalate, and 17 beta-estradiol have shared and unique dose-dependent effects on early embryo cleavage divisions and development in *Xenopus laevis*. Reprod Toxicol 84:65–74 10.1016/j.reprotox.2018.12.005 30579998 10.1016/j.reprotox.2018.12.005

[CR12] ASTM (2003) American Society for Testing and Materials, Standard guide for conducting the Frog Embryo Teragonesis Assay-*Xenopus* (FETAX), E1439-98. In: ASTM Standards on Biological Effects and Environmental Fate. Vol. 11.05. Philadelphia, PA, 447–457

[CR13] Aydin-Sinan H, Güngördü A, Ozmen M (2012) Toxic effects of deltamethrin and λ-cyhalothrin on *Xenopus laevis* tadpoles. J Environ Sci Heal 47:397–402 10.1080/03601234.2012.648545 10.1080/03601234.2012.648545 22424064

[CR15] Bonfanti P, Colombo A, Saibene M et al (2021) Microplastics from miscellaneous plastic wastes: Physico-chemical characterization and impact on fish and amphibian development. Ecotox Environ Safe 225:112775. 10.1016/j.ecoenv.2021.112775 10.1016/j.ecoenv.2021.112775 34536794

[CR16] Bradford MM (1976) A rapid and sensitive method for the quantitation of microgram quantities of protein utilizing the principle of protein-dye binding. Anal Biochem 72:248–254 10.1016/0003-2697(76)90527-3 942051 10.1016/0003-2697(76)90527-3

[CR18] Brander SM, Werner I, White JW et al (2009) Toxicity of a dissolved pyrethroid mixture to hyalella Azteca at environmentally relevant concentrations. Environ Toxicol Chem 28:1493–1499 10.1897/08-374.1 19249876 10.1897/08-374.1

[CR20] Burić P, Kovačić I, Jurković L et al (2023) Polymer chemical identity as a key factor in microplastic–insecticide antagonistic effects during embryogenesis of sea urchin *Arbacia lixula*. Int J Mol Sci 24:13 10.3390/ijms241310715 10.3390/ijms24044136 PMC9963837 36835548

[CR22] Cardoso-Vera JD, Islas-Flores H, SanJuan-Reyes N et al (2017) Comparative study of diclofenac-induced embryotoxicity and teratogenesis in *Xenopus laevis* and *Lithobates catesbeianus*, using the frog embryo teratogenesis assay: *Xenopus* (FETAX). Sci Total Environ 574:467–475 10.1016/j.scitotenv.2016.09.095 27644024 10.1016/j.scitotenv.2016.09.095

[CR24] Channing (1998) A Tadpoles as bio-indicators of stream quality: a baseline study. Water Research Commission of South Africa (WRC) Technical Report No. 718/1/98

[CR25] Cole M, Lindeque P, Halsband C et al (2011) Microplastics as contaminants in the marine environment: A review. Mar Pollut Bull 62:2588–2597. 10.1016/j.marpolbul.2011.09.025 22001295 10.1016/j.marpolbul.2011.09.025

[CR26] de Souza Freire I, Fascineli ML, Piau TB et al (2023) Multilevel toxicity evaluations of polyethylene microplastics in zebrafish (*Danio rerio*). Int J Environ Res Public HealthVol 20:3617 10.3390/ijerph20043617 10.3390/ijerph20043617 PMC9967563 36834310

[CR28] El-Demerdash FM (2007) Lambda-cyhalothrin-induced changes in oxidative stress biomarkers in rabbit erythrocytes and alleviation effect of some antioxidants. Toxicol Vitro 21:392–397 10.1016/j.tiv.2006.09.019 10.1016/j.tiv.2006.09.019 17137748

[CR30] Ellman GL, Courtney KD, Andres V Jr. et al (1961) A new and rapid colorimetric determination of acetylcholinesterase activity. Biochem Pharmacol 7:88–95 10.1016/0006-2952(61)90145-9 13726518 10.1016/0006-2952(61)90145-9

[CR32] Fang S, Yu W, Li C et al (2019) Adsorption behavior of three Triazole fungicides on polystyrene microplastics. Sci Total Environ 691:1119–1126 10.1016/j.scitotenv.2019.07.176 31466193 10.1016/j.scitotenv.2019.07.176

[CR34] Fernandez LP, Brasca R, Attademo AM et al (2020) Bioaccumulation and glutathione S-transferase activity on *Rhinella arenarum* tadpoles after short-term exposure to antiretrovirals. Chemosphere 246:125830 10.1016/j.chemosphere.2020.125830 31927383 10.1016/j.chemosphere.2020.125830

[CR36] Güngördü A, Turhan DO (2024) Biochemical studies to understand teratogenicity and lethality outcomes in modified-FETAX. In: Félix L (ed) Teratogenicity testing: methods and protocols. Springer US, New York, NY, pp 351–364 10.1007/978-1-0716-3625-1_19 38285350

[CR37] Habig WH, Pabst MJ, Jakoby WB (1974) Glutathione S-transferases. The first enzymatic step in mercapturic acid formation. J Biol Chem 249:7130–7139 4436300

[CR39] Hafsi D, Sbartai I, Sbartai H (2024) Stress biomarker response in *Aporrectodea caliginosa* earthworms exposed to single and combined pesticide treatments (Prosaro and Decis). Ecotoxicology 33:1180–1192 10.1007/s10646-024-02811-6 39379771 10.1007/s10646-024-02811-6

[CR41] Hasan AKMM, Hamed M, Hasan J et al (2024) A review of the neurobehavioural, physiological, and reproductive toxicity of microplastics in fishes. Ecotoxicol Environ Safe 282:116712 10.1016/j.ecoenv.2024.116712 10.1016/j.ecoenv.2024.116712 39002376

[CR43] He L-M, Troiano J, Wang A et al (2008) Environmental chemistry, ecotoxicity, and fate of lambda-cyhalothrin. In: Whitacre DM (ed) Reviews of environmental contamination and toxicology. Springer, New York, NY, pp 71–91 10.1007/978-0-387-77030-7_3 18418954

[CR44] Hong Y, Huang Y, Yan G et al (2021) DNA damage, immunotoxicity, and neurotoxicity induced by deltamethrin on the freshwater crayfish. Environ Toxicol 36:16–23. 10.1002/tox.23006 32757256 10.1002/tox.23006

[CR45] Horton AA, Vijver MG, Lahive E et al (2018) Acute toxicity of organic pesticides to *Daphnia magna* is unchanged by co-exposure to polystyrene microplastics. Ecotoxicol Environ Safe 166:26–34 10.1016/j.ecoenv.2018.09.052 10.1016/j.ecoenv.2018.09.052 30243044

[CR47] Houssini K, Li J, Tan Q (2025) Complexities of the global plastics supply chain revealed in a trade-linked material flow analysis. Commun Earth Environ 6:257. 10.1038/s43247-025-02169-5

[CR48] Kaushal J, Khatri M, Arya SK (2021) A treatise on organophosphate pesticide pollution: current strategies and advancements in their environmental degradation and elimination. Ecotoxicol Environ Safe 207:111483. 10.1016/j.ecoenv.2020.111483 10.1016/j.ecoenv.2020.111483 33120277

[CR49] Kim G-E, Kim D-W, Zee S (2024) Co-exposure to microplastic and plastic additives causes development impairment in zebrafish embryos. Aquat Toxicol 273:107001 10.1016/j.aquatox.2024.107001 38878329 10.1016/j.aquatox.2024.107001

[CR51] Kuder RS, Gundala HP (2018) Developmental toxicity of deltamethrin and 3-phenoxybenzoic acid in embryo–larval stages of zebrafish (*Danio rerio*). Toxicol Mech Methods 28:415–422 10.1080/15376516.2018.1439131 29421951 10.1080/15376516.2018.1439131

[CR53] Lajmanovich RC, Peltzer PM, Attademo AM et al (2019) First evaluation of novel potential synergistic effects of glyphosate and arsenic mixture on *Rhinella arenarum* (Anura: Bufonidae) tadpoles. Heliyon 5:e02601 10.1016/j.heliyon.2019.e02601 31687490 10.1016/j.heliyon.2019.e02601 PMC6820099

[CR55] Lee J-H, Kang J-C, Kim J-H (2023) Toxic effects of microplastic (Polyethylene) on fish: Accumulation, hematological parameters and antioxidant responses in Korean Bullhead, *Pseudobagrus Fulvidraco*. Sci Total Environ 877:162874 10.1016/j.scitotenv.2023.162874 36933717 10.1016/j.scitotenv.2023.162874

[CR57] Liu H, Wang WM, Zhang JF et al (2006) Effects of copper and its Ethylenediaminetetraacetate complex on the antioxidant defenses of the goldfish, *Carassius auratus*. Ecotoxicol Environ Safe 65:350–354. 10.1016/j.ecoenv.2005.09.002 10.1016/j.ecoenv.2005.09.002 16249031

[CR58] Lu Q, Sun Y, Ares I et al (2019) Deltamethrin toxicity: A review of oxidative stress and metabolism. Environ Res 170:260–281 10.1016/j.envres.2018.12.045 30599291 10.1016/j.envres.2018.12.045

[CR60] Luo T, Weng Y, Huang Z et al (2021) Combined hepatotoxicity of Imidacloprid and microplastics in adult zebrafish: endpoints at gene transcription. Comp Biochem Physiol C Toxicol Pharmacol 246:109043 10.1016/j.cbpc.2021.109043 33862234 10.1016/j.cbpc.2021.109043

[CR62] Malik AI, Storey KB (2009) Activation of antioxidant defense during dehydration stress in the African clawed frog. Gene 442:99–107 10.1016/j.gene.2009.04.007 19379800 10.1016/j.gene.2009.04.007

[CR64] Mann RM, Hyne RV, Choung CB et al (2009) Amphibians and agricultural chemicals: review of the risks in a complex environment. Environ Pollut 157:2903–2927. 10.1016/j.envpol.2009.05.015 19500891 10.1016/j.envpol.2009.05.015

[CR65] Mat Hussin SA, Varanusupakul P, Kassim MA et al (2021) Vortex assisted dispersive liquid–liquid Microextraction based on low transition temperature mixture solvent for the HPLC determination of pyrethroids in water samples: experimental study and COSMO-RS. Microchem J 171:106780 10.1016/j.microc.2021.106780

[CR67] Menegola E, Battistoni M, Bacchetta R et al (2024) Evaluation of temperature- and ethanol-related developmental degree variations by a new scoring system (FETAX-score) applicable to frog embryo teratogenicity assay: *Xenopus*. Reprod Toxicol 128:108632 10.1016/j.reprotox.2024.108632 38971262 10.1016/j.reprotox.2024.108632

[CR69] Meng Y, Kelly FJ, Wright SL (2020) Advances and challenges of microplastic pollution in freshwater ecosystems: A UK perspective. Environ Pollut 256:113445. 10.1016/j.envpol.2019.113445 31733965 10.1016/j.envpol.2019.113445

[CR70] Menon J, Rozman R (2007) Oxidative stress, tissue remodeling and regression during amphibian metamorphosis. Comp Biochem Physiol C Toxicol Pharmacol 145:625–631 10.1016/j.cbpc.2007.02.011 17395540 10.1016/j.cbpc.2007.02.011

[CR72] OECD (2009) Test No. 231: Amphibian Metamorphosis Assay. OECD Guidelines for the Testing of Chemicals, Sect. 2: Effects on Biotic Systems. OECD Publishing

[CR73] OECD (2019) Test Guideline No. 248: *Xenopus* Eleutheroembryo Thyroid Assay (XETA), Sect. 2: Effects on Biotic Systems. OECD Publishing

[CR74] Ozmen M, Dominguez SE, Fairbrother A (1998) Effects of dietary azinphos Methyl on selected plasma and tissue biomarkers of the gray-tailed vole. Bull Environ Contam Toxicol 60:194–201 10.1007/s001289900610 9470978 10.1007/s001289900610

[CR76] Peluso J, Gamarra F, Aronzon CM (2024) Synergistic interactions between the emerging contaminant Ivermectin and the ubiquitous pesticide glyphosate at an environmentally relevant ratio on *Rhinella arenarum* larvae. Chemosphere 358:142058. 10.1016/j.chemosphere.2024.142058 38642777 10.1016/j.chemosphere.2024.142058

[CR77] Pimpão CT, Zampronio AR, Silva de Assis HC (2007) Effects of deltamethrin on hematological parameters and enzymatic activity in *Ancistrus multispinis* (Pisces, Teleostei). Pestic Biochem Physiol 88:122–127. 10.1016/j.pestbp.2006.10.002

[CR78] PlasticsEurope (2023) Plastics Europe launches Plastics – the fast Facts 2023: https://plasticseurope.org/media/plastics-europe-launches-the-plastics-the-fast-facts-2023/

[CR79] Prokić MD, Gavrić JP, Petrović TG (2019) Oxidative stress in *Pelophylax esculentus* complex frogs in the wild during transition from aquatic to terrestrial life. Comp Biochem Physiol Mol Integr Physiol 234:98–105 10.1016/j.cbpa.2019.05.004 10.1016/j.cbpa.2019.05.004 31082485

[CR81] Rahman MM, Kim E-S, Sung H-C (2024) Microplastics as an emerging threat to amphibians: current status and future perspectives. Heliyon 10:e28220. 10.1016/j.heliyon.2024.e28220 38560268 10.1016/j.heliyon.2024.e28220 PMC10979166

[CR82] Ranatunga M, Kellar C, Pettigrove V (2023) Toxicological impacts of synthetic pyrethroids on non-target aquatic organisms: A review. Environ Adv 12:100388. 10.1016/j.envadv.2023.100388

[CR83] Ray DE, Fry JR (2006) A reassessment of the neurotoxicity of pyrethroid insecticides. Pharmacol Ther 111:174–193 10.1016/j.pharmthera.2005.10.003 16324748 10.1016/j.pharmthera.2005.10.003

[CR85] Relyea RA (2009) A cocktail of contaminants: how mixtures of pesticides at low concentrations affect aquatic communities. Oecologia 159:363–376. 10.1007/s00442-008-1213-9 19002502 10.1007/s00442-008-1213-9

[CR86] Ren Q, Zhang T, Li S et al (2016) Integrative characterization of toxic response of zebra fish (*Danio rerio*) to deltamethrin based on AChE activity and behavior strength. BioMed Res Int 2016:7309184 10.1155/2016/7309184 10.1155/2016/7309184 PMC5141558 27999812

[CR88] Rizzo AM, Adorni L, Montorfano G et al (2007) Antioxidant metabolism of *Xenopus laevis* embryos during the first days of development. Comp Biochem Physiol B Biochem Mol Biol 146:94–100 10.1016/j.cbpb.2006.09.009 17134930 10.1016/j.cbpb.2006.09.009

[CR90] Rodrigues S, Teixeira MI, Diogo BS et al (2023) Assessment of the ecotoxicological effects of deltamethrin to *Daphnia magna*: linking sub-individual and supra-individual parameters. Watershed Ecol Environ 5:231–240 10.1016/j.wsee.2023.10.002

[CR92] Salibián A (1992) Effects of deltamethrin on the South American toad, *Bufo arenarum*, tadpoles. Bull Environ Contam Toxicol 48:616–621 10.1007/bf00199082 1504507 10.1007/BF00199082

[CR94] Santhoshkumar P, Shivanandappa T (1999) In vitro sequestration of two organophosphorus homologs by the rat liver. Chem Biol Interact 119–120:277–282 10.1016/S0009-2797(99)00037-X 10.1016/s0009-2797(99)00037-x 10421462

[CR96] Satoh T, Hosokawa M (2006) Structure, function and regulation of carboxylesterases. Chem Biol Interact 162:195–211 10.1016/j.cbi.2006.07.001 16919614 10.1016/j.cbi.2006.07.001

[CR98] Shi T, Zhang Q, Chen X et al (2024) Overview of deltamethrin residues and toxic effects in the global environment. Environ Geochem Health 46:271. 10.1007/s10653-024-02043-x 38954040 10.1007/s10653-024-02043-x

[CR99] Soares MAM, Ferreira ERR, Tavares D et al (2024) Multi-biomarkers’ responses in gills of exposed to glyphosate and polyethylene microplastic, isolated and in mixture. Environ Toxicol 39:5048–5058 10.1002/tox.24386 39051743 10.1002/tox.24386

[CR101] Soderlund DM (2020) Neurotoxicology of pyrethroid insecticides. In: Aschner M, Costa, LG (eds) Advances in neurotoxicology, Academic Press, pp 113–165

[CR102] Srivastav AK, Srivastava SK, Srivastav SK (1997) Impact of deltamethrin on serum calcium and inorganic phosphate of freshwater catfish, *Heteropneustes fossilis*. Bull Environ Contam Toxicol 59:841–846 10.1007/s001289900558 9323238 10.1007/s001289900558

[CR104] Stephensen E, Svavarsson J, Sturve J et al (2000) Biochemical indicators of pollution exposure in shorthorn sculpin (*Myoxocephalus scorpius*), caught in four harbours on the Southwest Coast of Iceland. Aquat Toxicol 48:431–442 10.1016/S0166-445X(99)00062-4 10794829 10.1016/s0166-445x(99)00062-4

[CR106] Storck TR, Ames J, Qualhato G et al (2025) Differential biochemical responses of *Cyprinus Carpio* after dietary and waterborne exposure to microplastics from polyethylene-based biodegradable and conventional bags. 10.1007/s10646-025-02937-1. Ecotoxicology 10.1007/s10646-025-02937-1 40676486

[CR107] Subaramaniyam U, Allimuthu RS, Vappu S et al (2023) Effects of microplastics, pesticides and nano-materials on fish health, oxidative stress and antioxidant defense mechanism. Front Physiol 14:1217666 10.3389/fphys.2023.1217666 37435307 10.3389/fphys.2023.1217666 PMC10331820

[CR109] Sunny AR, Sazzad SA, Islam MA et al (2025) Microplastics in aquatic ecosystems: A global review of distribution, ecotoxicological impacts, and human health risks. Water 17:1741 10.3390/w17121741

[CR111] Tang KHD (2025) Combined toxicity of microplastics and antimicrobials on animals: A review. Antibiotics 14:896 10.3390/antibiotics14090896 41009875 10.3390/antibiotics14090896 PMC12466353

[CR113] Tatlı H, Gedik K, Altunışık A (2025) Distribution of microplastics in tadpoles, adults, and habitats of three water frogs of *Pelophylax spp*. Environ Sci Eur 37:27. 10.1186/s12302-025-01065-1

[CR114] Tierbach A, Groh KJ, Schonenberger R et al (2018) Glutathione S-transferase protein expression in different life stages of zebrafish (*Danio rerio*). Toxicol Sci 162:702–712 10.1093/toxsci/kfx293 29361160 10.1093/toxsci/kfx293 PMC5888913

[CR116] Timaná Morales M, Peraza Gómez V, Kozak ER et al (2025) Microplastics in marine fish: a mini-review on presence, classification, and impacts. Ecotoxicology 34:169–180. 10.1007/s10646-024-02837-w 39616298 10.1007/s10646-024-02837-w

[CR117] Trevisan R, Voy C, Chen S et al (2019) Nanoplastics decrease the toxicity of a complex PAH mixture but impair mitochondrial energy production in developing zebrafish. Environ Sci Technol 53:8405–8415 10.1021/acs.est.9b02003 31259535 10.1021/acs.est.9b02003 PMC6660138

[CR119] van der Oost R, Beyer J, Vermeulen NP (2003) Fish bioaccumulation and biomarkers in environmental risk assessment: a review. Environ Toxicol Pharmacol 13:57–149. 10.1016/S1382-6689(02)00126-6 21782649 10.1016/s1382-6689(02)00126-6

[CR120] Vijayavel K, Balasubramanian MP (2007) Interaction of potash and decis in the ecophysiology of a freshwater fish Oreochromis mossambicus. Ecotoxicol Environ Safe 66:154–158 10.1016/j.ecoenv.2005.12.005 10.1016/j.ecoenv.2005.12.005 16466792

[CR122] Wen B, Jin SR, Chen ZZ et al (2018) Single and combined effects of microplastics and cadmium on the cadmium accumulation, antioxidant defence and innate immunity of the discus fish (*Symphysodon aequifasciatus*). Environ Pollut 243:462–471. 10.1016/j.envpol.2018.09.029 30216878 10.1016/j.envpol.2018.09.029

[CR123] Wheelock CE, Shan G, Ottea J (2005) Overview of carboxylesterases and their role in the metabolism of insecticides. J Pestic Sci 30:75–83 10.1584/jpestics.30.75

[CR125] Xu K, Zhang Y, Huang Y et al (2021) Toxicological effects of microplastics and phenanthrene to zebrafish (*Danio rerio*). Sci Total Environ 757:143730 10.1016/j.scitotenv.2020.143730 33277007 10.1016/j.scitotenv.2020.143730

[CR127] Yadav RS, Sampath RR, Sharma VP (2001) Deltamethrin treatedb for control of malaria transmitted by *Anopheles culicifacies* (Diptera: Culicidae) in India. J Med Entomol 38:613–622 10.1603/0022-2585-38.5.613 11580032 10.1603/0022-2585-38.5.613

[CR129] Yang C, Lim W, Song G (2020) Mediation of oxidative stress toxicity induced by pyrethroid pesticides in fish. Comp Biochem Physiol C Toxicol Pharmacol 234:108758. 10.1016/j.cbpc.2020.108758 32289527 10.1016/j.cbpc.2020.108758

[CR130] Ye T, Yang R, He S (2025) Synergistic endocrine disruption and cellular toxicity of polyethylene microplastics and bisphenol A in MLTC-1 cells and zebrafish. Sci Rep 15:10752 10.1038/s41598-025-94902-5 40155689 10.1038/s41598-025-94902-5 PMC11953243

[CR132] Yu SY, Wages MR, Cai QS et al (2013) Lethal and sublethal effects of three insecticides on two developmental stages of *Xenopus laevis* and comparison with other amphibians. Environ Toxicol Chem 32:2056–2064 10.1002/etc.2280 23686650 10.1002/etc.2280

[CR134] Zhang K, Shi HH, Peng JP et al (2018) Microplastic pollution in china’s inland water systems: A review of findings, methods, characteristics, effects, and management. Sci Total Environ 630:1641–1653 10.1016/j.scitotenv.2018.02.300 29554780 10.1016/j.scitotenv.2018.02.300

[CR136] Zhang Q, Lv Y, Liu J et al (2023) Size matters either way: Differently-sized microplastics affect amphibian host and symbiotic microbiota discriminately. Environ Pollut 328:121634 10.1016/j.envpol.2023.121634 37054867 10.1016/j.envpol.2023.121634

