# Supplementary material for: The single and combined effects of deltamethrin and polyethylene microplastics on the development and biochemical responses of Xenopus laevis in early life stages
Source: Ecotoxicology. 2026 Jan 23;35(2):37. doi: 10.1007/s10646-026-03028-5 (PMC12827314; doi:10.1007/s10646-026-03028-5)
Supplement: Supplementary file 1 — Supplementary Material 1 [file 10646_2026_3028_MOESM1_ESM.docx]

**The Single and Combined Effects of Deltamethrin and Polyethylene Microplastics on the Development and Biochemical Responses of *Xenopus laevis* in Early Life Stages**

Duygu Ozhan Turhan, Cihan Anıl Benli, Muhittin Yurekli, Abbas Güngördü^*^

*Department of Biology, Faculty of Arts and Science, Inonu University, 44280 Malatya, Türkiye*

* Address correspondence to A. Güngördü, Laboratory of Environmental Toxicology, *Department of Biology*, Inonu University, Faculty of Arts and Science, Malatya, 44280, Türkiye

Email: [abbas.gungordu@inonu.edu.tr](mailto:abbas.gungordu@inonu.edu.tr)

**Supplementary Material**

**Statistical Power Analysis**

For each biomarker assay (GST, GR, CAT, CaE, AChE), five independent replicates were analyzed per treatment, each consisting of a pooled sample of 15 embryos or tadpoles. Power analysis was conducted a priori (α = 0.05, two-tailed) using the observed coefficients of variation (CVs) in the control groups to ensure adequate sensitivity across endpoints.

In embryos, CVs ranged between approximately 5–26%, whereas in tadpoles they ranged between 3–26%. Under these conditions, the present design (n = 5 per treatment) yields ≥80% statistical power to detect large effects corresponding to Cohen’s d ≈ 1.76 (≈25–35% mean change). The minimum detectable difference (MDE, % change at 80% power) varied between ~9–45% for embryos and ~5–45% for tadpoles depending on biomarker variability.

In contrast, morphometric parameters such as body length exhibited much lower variability (CV ≈ 4–7%, n = 46–48 per group), providing >80% power to detect small (~3–4%) mean changes. Consequently, the experimental design ensured sufficient sensitivity for developmental endpoints, whereas subtle biochemical responses at low concentrations—especially for variable markers such as GR, CAT, and AChE—may remain undetected (Type II error risk).

Power and effect-size calculations were based on empirical CVs and standard two-tailed test assumptions.

**Table S1.** Characterization of polyethylene microplastics (PE-MPs, 100 mg/L) alone and in combination with deltamethrin (DEL, 13.6 µg/L) in FETAX medium. Particle size was measured under light microscopy (400×) and analyzed in GraphPad Prism 8. Values are presented as mean ± SD, median, minimum, maximum, and percentiles.

| **Parameter** | **PE-MP (100 mg/L)** | **DEL/PE-MP (13.6** **µg/L +100 mg/L)** |
| --- | --- | --- |
| n (number of particles) | 105 | 105 |
| Mean diameter (µm) | 41.40±11.24 | 39.86±13.50 |
| Median (µm) | 42.06 | 36.92 |
| Minimum (µm) | 16.94 | 19.21 |
| Maximum (µm) | 77.20 | 74.08 |
| 10 % Percentile | 26.34 | 25.23 |
| 90 % Percentile | 55.15 | 60.98 |

**Table S2.** Nominal and measured concentrations of deltamethrin in the exposure media prior to FETAX testing

| **Groups** | **Concentration** | | | | |
| --- | --- | --- | --- | --- | --- |
|  | **Nominal (µg/L)** | | **Measured (µg/L)** | | |
| **DEL** | LC_50_/25 | 2.72 | 2.42 | ± | 0.13 |
|  | LC_50_/5 | 13.6 | 15.35 | ± | 0.79 |
| **DEL/PE-MP** | LC_50_/25 | 2.72 | 2.47 | ± | 0.63 |
|  | LC_50_/5 | 13.6 | 19.96 | ± | 2.34 |

**Table S3.** Time-dependent toxicity data in *X. laevis* embryos exposed to different DEL concentrations.

|  |  |  | | | | **FETAX details after 96 h** | | | | | | | | | | | | | | |
| --- | --- | --- | --- | --- | --- | --- | --- | --- | --- | --- | --- | --- | --- | --- | --- | --- | --- | --- | --- | --- |
|  |  | **Lethality** | | | | **Lived** | **Malformed** | **Types of Malformations #(%)** | | | | | | | | |  | | | |
| **Conc. (µg/L)** | ***n*** | **24 *h*** | **48 *h*** | **72 *h*** | **96 *h*** | **#(%)** | **#(%)** | **Sv** | **St** | **Gut** | **AE** | **CFE** | **TC** | **Mc** | **Mf** | **Bl** | **Length (mm)** | | | |
| Control | 48 | 0 | 1 | 1 | 1 | 47 (98) | 2 (4) |  |  | 1 (2) |  |  | 1 (2) |  |  |  | 7.62 | ± | 0.052 |  |
| DMSO-Control | 48 | 0 | 1 | 1 | 2 | 46 (96) | 2 (4) |  | 1 (2) | 2 (4) | 2 (4) | 1 (2) | 1 (2) |  |  |  | 7.55 | ± | 0.068 |  |
| 3.125 | 32 | 0 | 1 | 1 | 1 | 31 (97) | 3 (10) |  |  |  |  |  | 3 (10) |  |  |  | 7.35 | ± | 0.060 |  |
| 6.25 | 32 | 0 | 0 | 0 | 1 | 31 (97) | 9 (29) |  |  |  |  |  | 9 (29) |  |  |  | 6.71 | ± | 0.068 | *** |
| 12.5 | 32 | 0 | 1 | 1 | 3 | 29 (91) | 28 (97) | 1 (3) | 1 (3) | 9 (31) | 3 (10) | 4 (14) | 27 (93) |  | 1 (3) |  | 6.39 | ± | 0.109 | *** |
| 25 | 32 | 0 | 0 | 1 | 14 | 18 (56) | 18 (100) |  | 16 (89) | 16 (89) | 1 (6) |  | 17 (94) |  | 1 (6) |  | 5.64 | ± | 0.118 | *** |
| 50 | 32 | 0 | 0 | 1 | 16 | 16 (50) | 16 (100) | 3 (19) | 9 (56) | 13 (81) | 4 (25) | 3 (19) | 16 (100) |  | 1 (6) |  | 5.47 | ± | 0.169 | *** |
| 100 | 32 | 0 | 0 | 0 | 20 | 12 (38) | 12 (100) |  | 4 (33) | 10 (83) |  | 3 (25) | 10 (83) |  |  |  | 5.92 | ± | 0.136 | *** |
| 200 | 32 | 0 | 2 | 2 | 21 | 11 (34) | 11 (100) | 7 (64) | 11 (100) | 11 (100) |  |  | 11 (100) |  |  |  | 5.10 | ± | 0.118 | *** |
| 400 | 32 | 0 | 0 | 11 | 27 | 5 (16) | 5 (100) | 4 (80) | 5 (100) | 5 (100) |  |  | 5 (100) |  |  |  | 3.96 | ± | 0.165 | *** |
| 800 | 32 | 0 | 0 | 11 | 29 | 3 (9) | 3 (100) | 3 (100) | 3 (100) | 3 (100) | 3 (100) | 2 (67) | 3 (100) |  |  |  | 4.14 | ± | 0.330 | *** |
| 1600 | 32 | 0 | 0 | 16 | 32 | 0 | - |  |  |  |  |  |  |  |  |  |  |  |  |  |
| 3200 | 32 | 0 | 0 | 22 | 32 | 0 | - |  |  |  |  |  |  |  |  |  |  |  |  |  |
| 6400 | 32 | 0 | 0 | 19 | 32 | 0 | - |  |  |  |  |  |  |  |  |  |  |  |  |  |

*n*: Each control group consisted of 48 individuals (4 per replicate, 12 replicates), while each treatment group included 32 individuals (4 per replicate, 8 replicates).

Malformation Types: Sv: Severe (multiple, severe malformations), St: Stunted growth, Gut: gut abnormalities (improper gut coiling, long loosely coiled gut), AE: Abdominal edema, CFE: Craniofacial edema (face, head and eye edema), Mc: Microcephaly, Mf: Microphthalmia, TC: Tail curvature, Bl: Blister

Data are expressed as mean ± SE. Statistical analysis was performed using one-way ANOVA followed by Dunnett’s multiple-comparison test or Kruskal-Wallis, followed by Dunn’s test.

*, ** and ***: Significant compared to control group at p < 0.05, p < 0.01 and p < 0.001, respectively.

**Table S4.** Time-dependent toxicity data in *X. laevis* embryos exposed to different PE-MP concentrations.

|  |  |  | | | | **FETAX details after 96 h** | | | | | | | | | | | | | | |
| --- | --- | --- | --- | --- | --- | --- | --- | --- | --- | --- | --- | --- | --- | --- | --- | --- | --- | --- | --- | --- |
|  |  | **Lethality** | | | | **Lived** | **Malformed** | **Types of Malformations #(%)** | | | | | | | | |  | | | |
| **Conc. (mg/L)** | ***n*** | **24 *h*** | **48 *h*** | **72 *h*** | **96 *h*** | **#(%)** | **#(%)** | **Sv** | **St** | **Gut** | **AE** | **CFE** | **TC** | **Mc** | **Mf** | **Bl** | **Length (mm)** | | | |
| Control | 48 | 0 | 1 | 1 | 1 | 47 (98) | 2 (4) |  |  | 1 (2) |  |  | 1 (2) |  |  |  | 7.62 | ± | 0.052 |  |
| 50 | 32 | 0 | 0 | 1 | 1 | 31 (97) | 1 (3) |  | 1 (3) | 1 (3) | 1 (3) |  | 1 (3) |  |  |  | 7.44 | ± | 0.069 |  |
| 100 | 32 | 0 | 0 | 0 | 0 | 32 (100) | 1 (3) |  | 1 (3) |  |  |  |  |  |  |  | 7.55 | ± | 0.067 |  |
| 250 | 32 | 0 | 1 | 1 | 1 | 31 (97) | 1 (3) |  | 1 (3) |  |  | 1 (3) | 1 (3) |  |  |  | 7.54 | ± | 0.063 |  |
| 500 | 32 | 0 | 1 | 1 | 1 | 31 (97) | 2 (6) |  | 1 (3) | 1 (3) | 1 (3) | 2 (6) | 1 (3) |  |  |  | 7.33 | ± | 0.084 | * |
| 1000 | 32 | 0 | 0 | 0 | 0 | 32 (100) | 4 (13) |  |  |  |  |  | 4 (13) |  |  |  | 7.46 | ± | 0.070 |  |

*n*: Each control group consisted of 48 individuals (4 per replicate, 12 replicates), while each treatment group included 32 individuals (4 per replicate, 8 replicates).

Malformation Types: Sv: Severe (multiple, severe malformations), St: Stunted growth, Gut: gut abnormalities (improper gut coiling, long loosely coiled gut), AE: Abdominal edema, CFE: Craniofacial edema (face, head and eye edema), Mc: Microcephaly, Mf: Microphthalmia, TC: Tail curvature, Bl: Blister

Data are expressed as mean ± SE. Statistical analysis was performed using one-way ANOVA followed by Dunnett’s multiple-comparison test or Kruskal-Wallis, followed by Dunn’s test.

*, ** and ***: Significant compared to control group at p < 0.05, p < 0.01 and p < 0.001, respectively.

**Table S5.** Time-dependent toxicity data for *X. laevis* embryos exposed to sublethal DEL and PE-MP concentrations alone and DEL/PE-MP mixtures.

|  |  | |  |  | | | | **FETAX details after 96 h** | | | | | | | | | | | | | | |
| --- | --- | --- | --- | --- | --- | --- | --- | --- | --- | --- | --- | --- | --- | --- | --- | --- | --- | --- | --- | --- | --- | --- |
|  |  | |  | **Lethality** | | | | **Lived** | **Malformed** | **Types of Malformations #(%)** | | | | | | | | |  | | | |
| **Groups** | **Concentration** | | ***n*** | **24 *h*** | **48 *h*** | **72 *h*** | **96 *h*** | **#(%)** | **#(%)** | **Sv** | **St** | **Gut** | **AE** | **CFE** | **TC** | **Mc** | **Mf** | **Bl** | **Length (mm)** | | | |
|  | **DEL (µg/L)** | **PE-MP (mg/L)** |  |  |  |  |  |  |  |  |  |  |  |  |  |  |  |  |  |  |  |  |
| **Control** | – | – | 48 | 0 | 1 | 1 | 1 | 47 (98) | 3 (6) |  |  | 2(4) | 2(4) |  | 2(4) |  | 1(2) |  | 7.32 | ± | 0.072 |  |
| **DMSO** | – | – | 48 | 1 | 1 | 1 | 1 | 47 (98) | 4 (9) | 1(2) | 3(6) | 3(6) | 3(6) | 1(2) | 4(9) |  | 1(2) |  | 7.25 | ± | 0.097 |  |
| **DEL** | 2.72 | – | 32 | 1 | 4 | 4 | 4 | 28 (88) | 3 (11) |  | 2(7) | 3(11) |  | 1(4) | 1(4) |  |  |  | 7.48 | ± | 0.093 |  |
|  | 13.6 | – | 32 | 1 | 2 | 2 | 2 | 30 (94) | 14 (47) |  | 1(3) | 6(20) | 4(13) | 3(10) | 13(43) |  | 1(3) |  | 6.88 | ± | 0.132 | * |
| **DEL/PE-MP** | 2.72 | 100 | 32 | 0 | 0 | 0 | 0 | 32 (100) | 6 (19) | 1(3) | 3(9) | 5(16) | 3(9) | 2(6) | 4(13) | 1(3) | 3(9) |  | 7.31 | ± | 0.093 |  |
|  | 13.6 | 100 | 32 | 0 | 0 | 0 | 0 | 32 (100) | 15 (47) | 2(6) | 5(16) | 6(19) | 2(6) | 3(9) | 15(47) | 3(9) | 3(9) | 1(3) | 6.95 | ± | 0.145 | *^a^* |
| **PE-MP** | – | 50 | 32 | 2 | 3 | 3 | 3 | 29 (91) | 4 (14) | 3(10) | 3(10) | 4(14) | 3(10) | 2(7) | 4(14) | 3(10) | 3(10) |  | 7.47 | ± | 0.164 | * |
|  | – | 100 | 32 | 0 | 0 | 1 | 1 | 31 (97) | 3 (10) |  | 1(3) | 1(3) | 1(3) | 1(3) | 2(6) |  | 1(3) |  | 7.54 | ± | 0.073 | *^a^* |
|  | – | 250 | 32 | 0 | 1 | 1 | 1 | 31 (97) | 4 (13) |  | 2(6) | 3(10) | 2(6) |  | 2(6) |  | 1(3) |  | 7.51 | ± | 0.098 |  |

*n*: Each control group consisted of 48 individuals (4 per replicate, 12 replicates), while each treatment group included 32 individuals (4 per replicate, 8 replicates).

Malformation Types: Sv: Severe (multiple, severe malformations), St: Stunted growth, Gut: gut abnormalities (improper gut coiling, long loosely coiled gut), AE: Abdominal edema, CFE: Craniofacial edema (face, head and eye edema), Mc: Microcephaly, Mf: Microphthalmia, TC: Tail curvature, Bl: Blister

Data are expressed as mean ± SE. Statistical analysis was performed using one-way ANOVA followed by Dunnett’s multiple-comparison test or Kruskal-Wallis, followed by Dunn’s test.

*, ** and ***: Significant compared to control group at p < 0.05, p < 0.01 and p < 0.001, respectively.

The letter a indicates a statistically significant difference (p < 0.05) based on the Mann–Whitney U test.

**Table S6.** The biomarker enzyme activities in *X. laevis* embryos exposed to sublethal DEL and PE-MP concentrations alone and DEL/PE-MP mixtures.

| **Groups** | **Concentration** | | ***n*** | **GST** | | |  | **GR** | | |  | **CAT** | | |  | **CaE** | | |  | **AChE** | | |  |
| --- | --- | --- | --- | --- | --- | --- | --- | --- | --- | --- | --- | --- | --- | --- | --- | --- | --- | --- | --- | --- | --- | --- | --- |
|  | **DEL (µg/L)** | **PE-MP (mg/L)** |  |  |  |  |  |  |  |  |  |  |  |  |  |  |  |  |  |  |  |  |  |
| **Control** | – | – | 5 | 200.9 | ± | 6.23 |  | 5.09 | ± | 0.59 |  | 9.20 | ± | 0.68 |  | 150.4 | ± | 3.65 |  | 9.56 | ± | 0.76 |  |
| **DMSO** | – | – | 5 | 199.8 | ± | 12.22 |  | 4.99 | ± | 0.43 |  | 8.88 | ± | 0.75 |  | 150.7 | ± | 7.12 |  | 9.89 | ± | 0.60 |  |
| **DEL** | 2.72 | – | 5 | 239.8 | ± | 9.87 | * | 4.57 | ± | 0.40 |  | 11.15 | ± | 0.91 | *^a^* | 172.2 | ± | 3.37 | * | 12.28 | ± | 0.86 | * |
|  | 13.6 | – | 5 | 241.7 | ± | 12.35 | * | 4.54 | ± | 0.32 |  | 11.62 | ± | 0.83 | *^b^* | 169.0 | ± | 6.27 |  | 12.90 | ± | 0.42 | *** |
| **DEL/PE-MP** | 2.72 | 100 | 5 | 241.7 | ± | 8.10 | * | 4.16 | ± | 0.30 |  | 15.11 | ± | 0.91 | *^,^*^a^* | 204.4 | ± | 16.8 | ** | 11.99 | ± | 0.91 |  |
|  | 13.6 | 100 | 5 | 257.4 | ± | 9.76 | ** | 3.76 | ± | 0.34 |  | 26.66 | ± | 2.97 | ****^,b,c^* | 166.3 | ± | 3.61 |  | 15.54 | ± | 1.19 | ** |
| **PE-MP** | – | 50 | 5 | 223.1 | ± | 8.21 |  | 3.42 | ± | 0.50 | * | 16.57 | ± | 2.79 | * | 144.6 | ± | 8.9 |  | 14.14 | ± | 1.07 | * |
|  | – | 100 | 5 | 217.4 | ± | 15.84 |  | 3.86 | ± | 0.39 |  | 15.34 | ± | 1.01 | **^,c^* | 186.8 | ± | 20.01 |  | 14.06 | ± | 0.48 | * |
|  | – | 250 | 5 | 239.2 | ± | 6.12 | * | 3.44 | ± | 0.24 | * | 14.18 | ± | 1.35 |  | 168.3 | ± | 6.95 |  | 11.55 | ± | 0.12 |  |

*n:* Each group consisted of 75 individuals (15 per replicate, 5 replicates).

All enzyme activities are expressed as nmol min⁻¹ mg⁻¹ protein ± standard error.

Statistical analyses were performed using one-way ANOVA followed by Dunnett’s multiple comparison test, or Kruskal–Wallis test followed by Dunn’s post hoc test, depending on data distribution.

*, **, and ***: Significantly different from the control group at p < 0.05, p < 0.01, and p < 0.001, respectively.

Letters indicate statistically significant pairwise differences (p < 0.05) based on independent t-tests (parametric) or Mann–Whitney U tests (nonparametric) between the groups marked with the same letter (*a–c*).

**Table S7.** The biomarker enzyme activities in *X. laevis* tadpoles exposed to sublethal DEL and PE-MP concentrations alone and DEL/PE-MP mixtures.

| **Groups** | **Concentration** | | ***n*** | **GST** | | |  | **GR** | | |  | **CAT** | | |  | **CaE** | | |  | **AChE** | | |  |
| --- | --- | --- | --- | --- | --- | --- | --- | --- | --- | --- | --- | --- | --- | --- | --- | --- | --- | --- | --- | --- | --- | --- | --- |
|  | **DEL (µg/L)** | **PE-MP (mg/L)** |  |  |  |  |  |  |  |  |  |  |  |  |  |  |  |  |  |  |  |  |  |
| **Control** | – | – | 5 | 274.0 | ± | 3.94 |  | 9.37 | ± | 0.50 |  | 6.51 | ± | 0.75 |  | 232.0 | ± | 10.99 |  | 126.1 | ± | 6.88 |  |
| **DMSO** | – | – | 5 | 275.8 | ± | 19.48 |  | 9.19 | ± | 0.48 |  | 8.14 | ± | 0.81 |  | 237.3 | ± | 11.33 |  | 108.4 | ± | 7.06 |  |
| **DEL** | 2.72 | – | 5 | 377.0 | ± | 14.13 | ** | 7.12 | ± | 0.42 |  | 12.08 | ± | 0.91 | **^,a^* | 301.4 | ± | 7.56 | *** | 115.7 | ± | 3.69 |  |
|  | 13.6 | – | 5 | 393.1 | ± | 23.24 | *** | 7.54 | ± | 0.72 |  | 12.14 | ± | 2.05 | * | 238.0 | ± | 10.16 |  | 138.2 | ± | 10.70 |  |
| **DEL/PE-MP** | 2.72 | 100 | 5 | 364.0 | ± | 15.91 | ***^,a^* | 8.47 | ± | 0.55 |  | 7.86 | ± | 1.04 | *^a^* | 277.6 | ± | 12.69 | **^,a^* | 129.2 | ± | 9.43 |  |
|  | 13.6 | 100 | 5 | 340.5 | ± | 14.83 | **^,b^* | 9.85 | ± | 0.78 |  | 8.36 | ± | 1.46 |  | 232.7 | ± | 5.63 |  | 122.6 | ± | 7.44 |  |
| **PE-MP** | – | 50 | 5 | 258.9 | ± | 17.10 |  | 10.21 | ± | 1.17 |  | 8.06 | ± | 1.32 |  | 225.9 | ± | 13.40 |  | 114.8 | ± | 2.91 |  |
|  | – | 100 | 5 | 289.5 | ± | 8.39 | *^a,b^* | 7.15 | ± | 1.12 |  | 7.25 | ± | 0.57 |  | 235.2 | ± | 7.11 | *^a^* | 107.8 | ± | 2.94 |  |
|  | – | 250 | 5 | 283.3 | ± | 4.31 |  | 5.67 | ± | 0.49 | * | 8.03 | ± | 0.96 |  | 215.9 | ± | 10.41 |  | 89.2 | ± | 4.06 | ** |

*n:* Each group consisted of 75 individuals (15 per replicate, 5 replicates).

All enzyme activities are expressed as nmol min⁻¹ mg⁻¹ protein ± standard error.

Statistical analyses were performed using one-way ANOVA followed by Dunnett’s multiple comparison test, or Kruskal–Wallis test followed by Dunn’s post hoc test, depending on data distribution.

*, **, and ***: Significantly different from the control group at p < 0.05, p < 0.01, and p < 0.001, respectively.

Letters indicate statistically significant pairwise differences (p < 0.05) based on independent t-tests (parametric) or Mann–Whitney U tests (nonparametric) between the groups marked with the same letter (*a–c*).


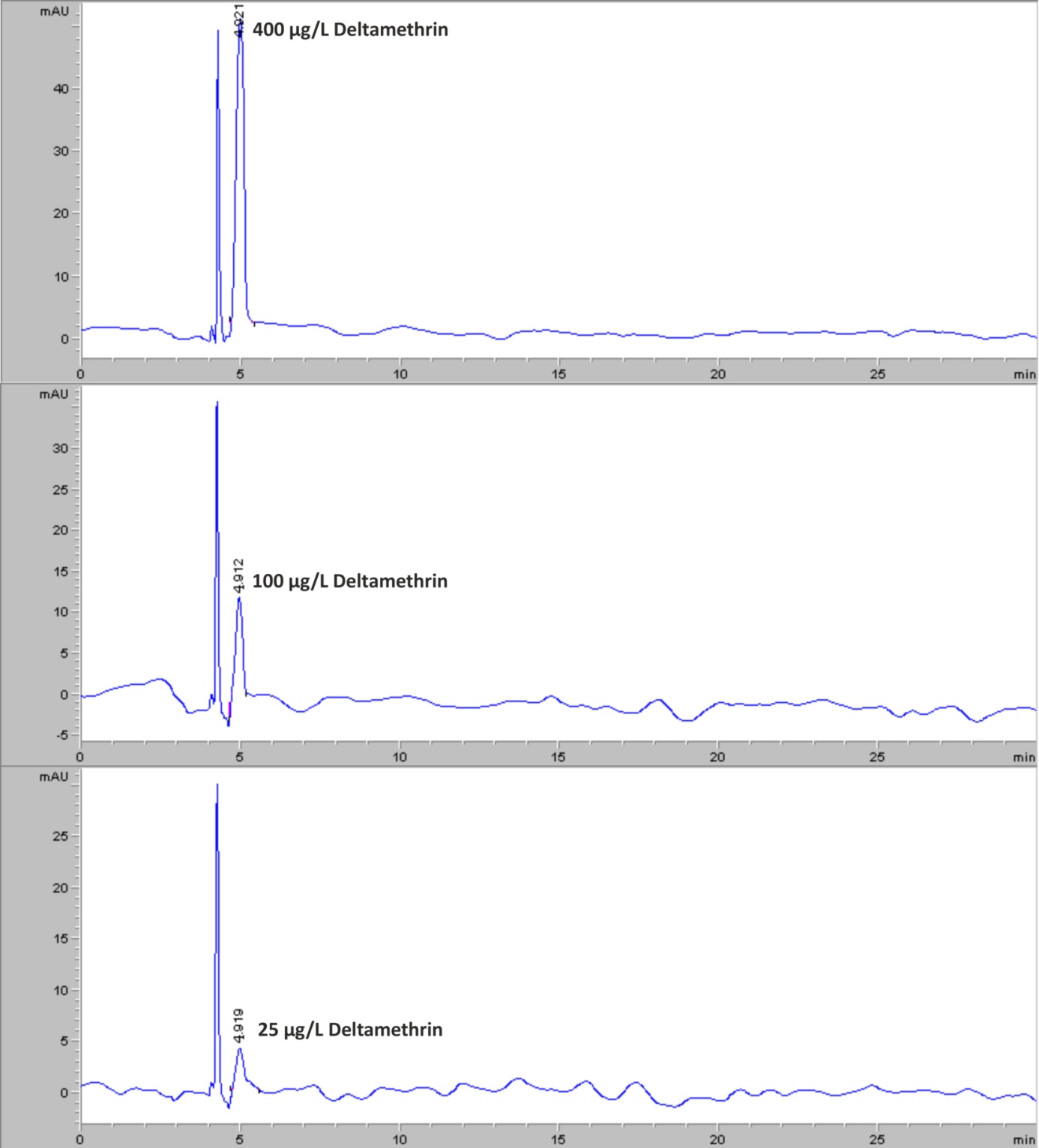


**Figure S1.** The HPLC chromatograms of deltamethrin

**
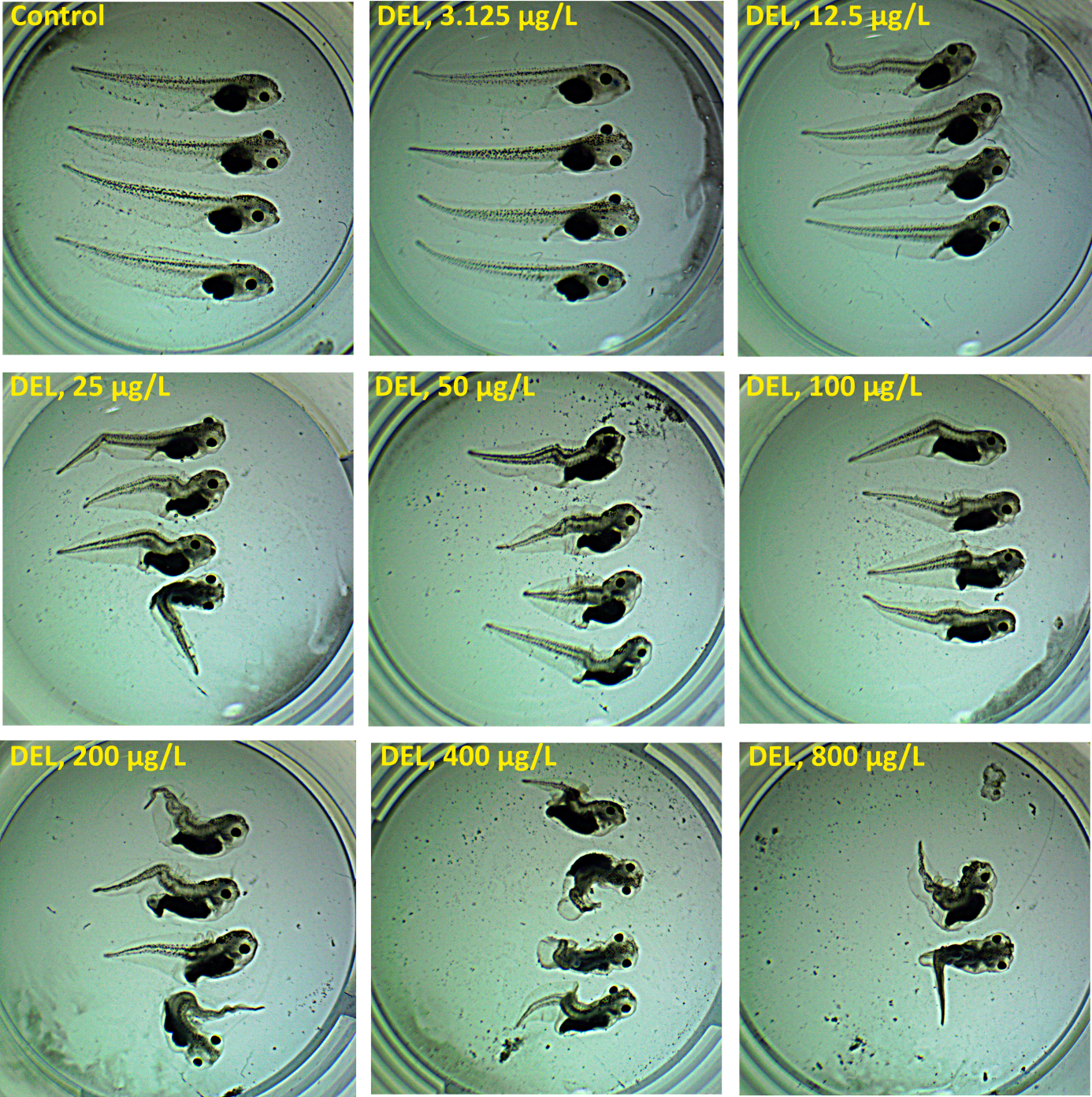
**

**Figure S2.** Appearance of *Xenopus laevis* embryos exposed to different DEL concentrations (3.125–800 µg/L).


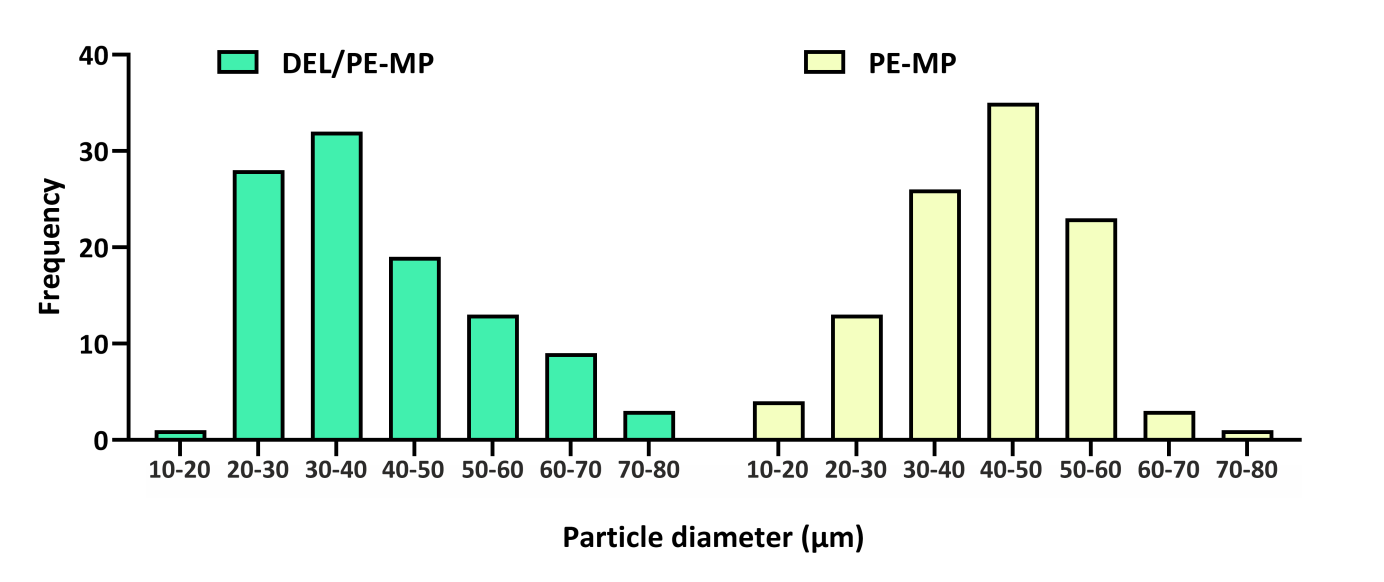


**Figure S3.** Histogram of particle size distribution of polyethylene microplastics (PE-MPs, 100 mg/L, n = 105) and PE-MPs in combination with deltamethrin (100 mg/L PE-MPs + 13.6 µg/L DEL, n = 105). Particle diameters were measured under light microscopy and binned in 10 µm intervals.

**
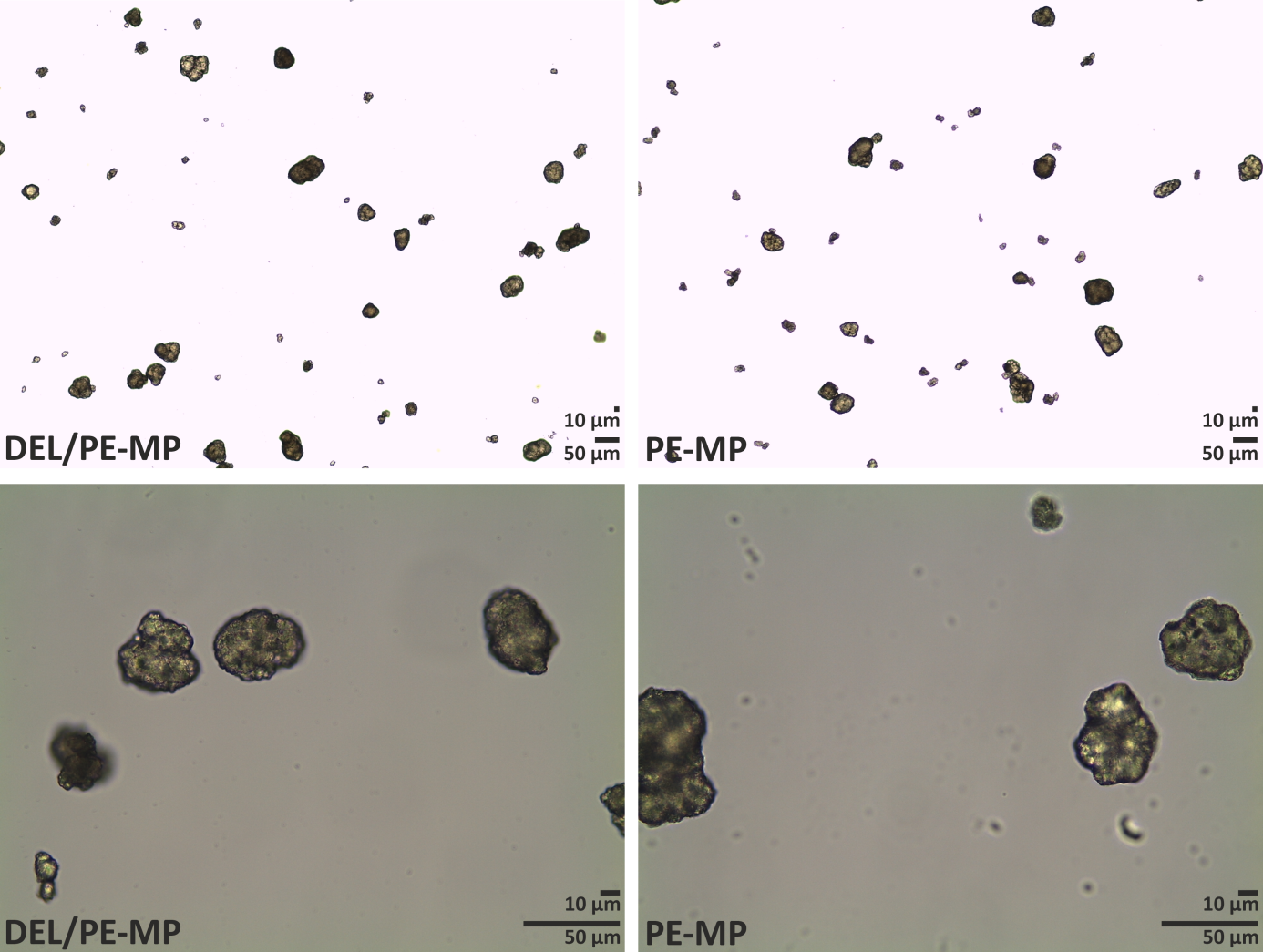
**

**Figure S4.** Representative light microscopy images of polyethylene microplastics (PE-MPs, 100 mg/L) alone (right panels) and in combination with deltamethrin (DEL/PE-MP; 100 mg/L PE-MPs + 13.6 µg/L DEL; left panels). Upper panels show dispersed particles, while lower panels show close-up views highlighting morphology. Scale bars (shown in each panel): upper bar = 10 µm; lower bar = 50 µm.


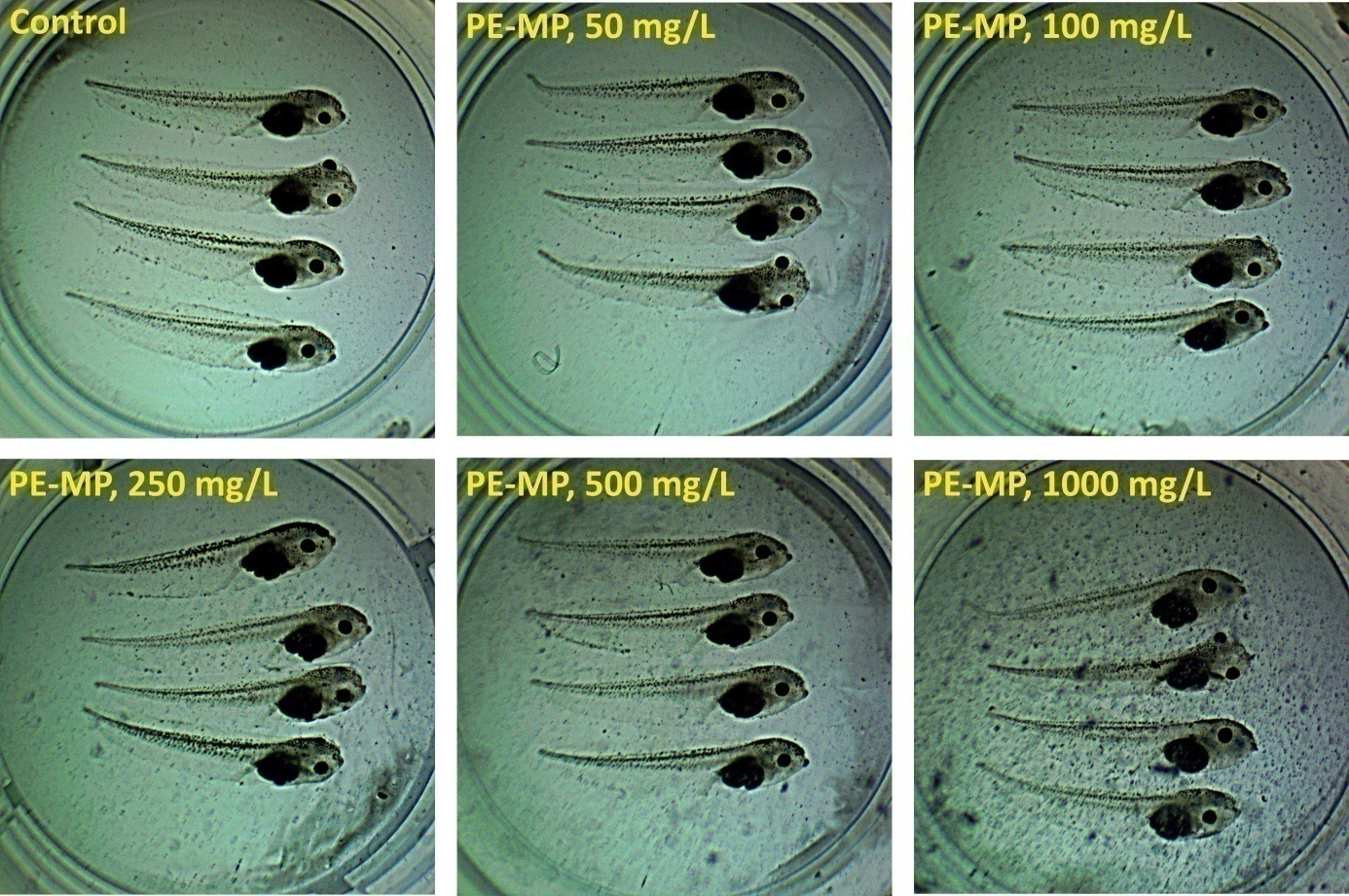


**Figure S5.** Appearance of *Xenopus laevis* embryos exposed to different PE-MP concentrations (50–1000 mg/L).


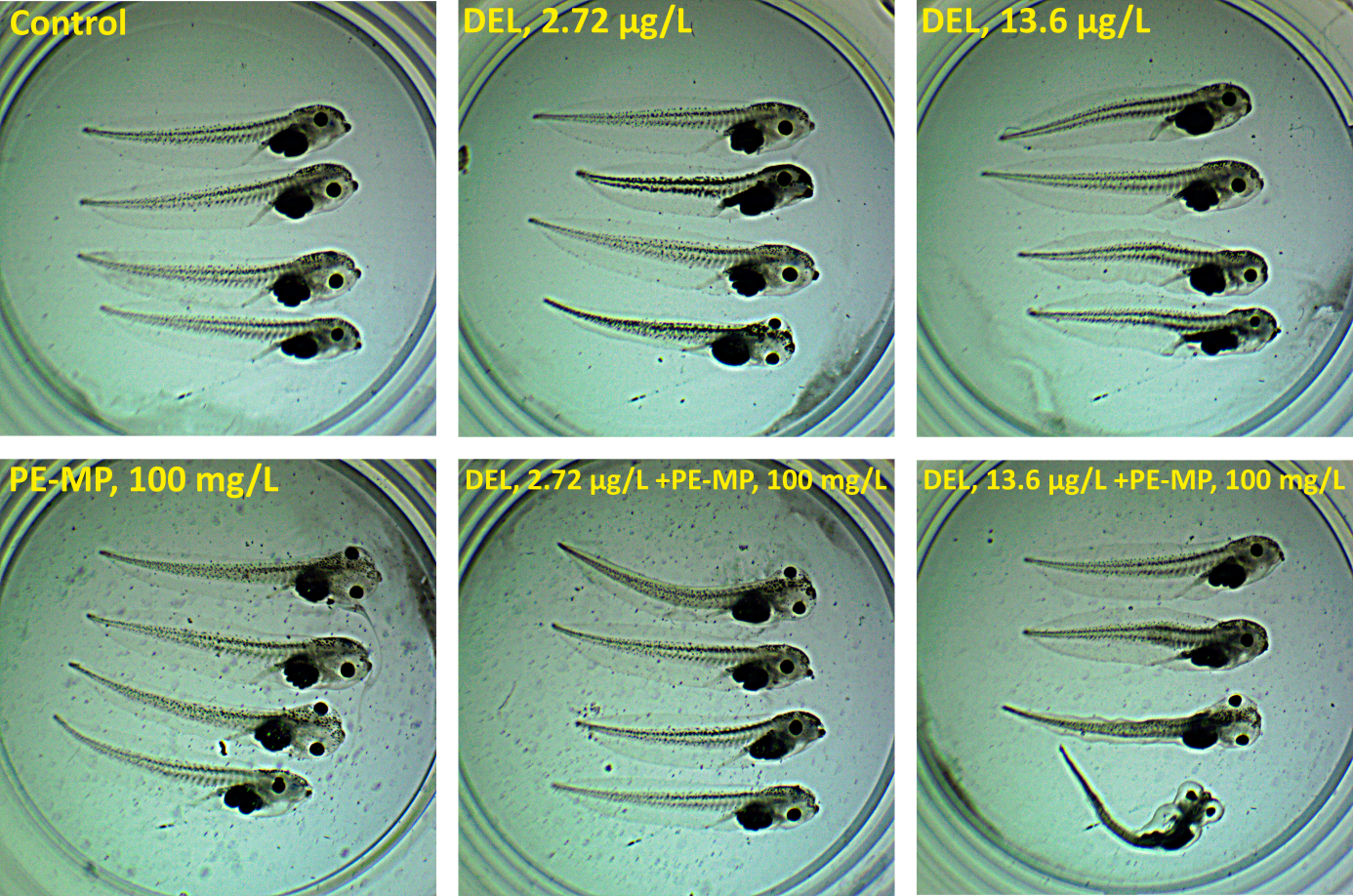


**Figure S6.** Appearance of *Xenopus laevis* embryos exposed to sublethal DEL concentrations (2.72 and 13.6 µg/L), PE-MPs (100 mg/L) alone, and DEL/PE-MP mixtures.
